# The LRR receptor-like kinase ALR1 is a plant aluminum ion sensor

**DOI:** 10.1038/s41422-023-00915-y

**Published:** 2024-01-10

**Authors:** Zhong Jie Ding, Chen Xu, Jing Ying Yan, Yu Xuan Wang, Meng Qi Cui, Jun Jie Yuan, Ya Nan Wang, Gui Xin Li, Jian Xiang Wu, Yun Rong Wu, Ji Ming Xu, Chun Xiao Li, Yuan Zhi Shi, Chuan Zao Mao, Jiang Tao Guo, Jian Min Zhou, Moussa Benhamed, Nicholas P. Harberd, Shao Jian Zheng

**Affiliations:** 1 State Key Laboratory of Plant Environmental Resilience, College of Life Sciences, Zhejiang University, Hangzhou, Zhejiang China; 2 Guangdong Laboratory for Lingnan Modern Agriculture, College of Natural Resources and Environment, South China Agricultural University, Guangzhou, Guangdong China; 3 Agricultural Experimental Station, Zhejiang University, Hangzhou, Zhejiang China; 4 State Key Laboratory of Rice Biology, Institute of Biotechnology, Zhejiang University, Hangzhou, Zhejiang China; 5 Tea Research Institute, Chinese Academy of Agricultural Sciences, Hangzhou, Zhejiang China; 6 Medical School, Zhejiang University, Hangzhou, Zhejiang China; 7 Center for Genome Biology and State Key Laboratory of Plant Genomics, Institute of Genetics and Developmental Biology, Chinese Academy of Sciences, Beijing, China; 8 Université Paris-Saclay, CNRS, INRAE, Univ Evry, Institute of Plant Sciences Paris-Saclay (IPS2), Orsay, France; 9 Department of Biology, University of Oxford, Oxford, UK; 10 Institute of Ecological Civilization, Zhejiang University, Hangzhou, Zhejiang China

**Keywords:** Plant signalling, Plant molecular biology

## Abstract

Plant survival requires an ability to adapt to differing concentrations of nutrient and toxic soil ions, yet ion sensors and associated signaling pathways are mostly unknown. Aluminum (Al) ions are highly phytotoxic, and cause severe crop yield loss and forest decline on acidic soils which represent ∼30% of land areas worldwide. Here we found an *Arabidopsis* mutant hypersensitive to Al. The gene encoding a leucine-rich-repeat receptor-like kinase, was named Al Resistance1 (ALR1). Al ions binding to ALR1 cytoplasmic domain recruits BAK1 co-receptor kinase and promotes ALR1-dependent phosphorylation of the NADPH oxidase RbohD, thereby enhancing reactive oxygen species (ROS) generation. ROS in turn oxidatively modify the RAE1 F-box protein to inhibit RAE1-dependent proteolysis of the central regulator STOP1, thus activating organic acid anion secretion to detoxify Al. These findings establish ALR1 as an Al ion receptor that confers resistance through an integrated Al-triggered signaling pathway, providing novel insights into ion-sensing mechanisms in living organisms, and enabling future molecular breeding of acid-soil-tolerant crops and trees, with huge potential for enhancing both global food security and forest restoration.

## Introduction

Aluminum (Al) is the most abundant metal in the Earth’s crust (∼8% by weight). However, the Al ion is highly toxic to plants. When soils become acidic, part of Al is solubilized from insoluble aluminosilicates or oxides to form soluble ions.^1^ The resultant phytotoxic Al ions can rapidly enter root cells, and cause a series of cellular damages,^2^ thus inhibiting root growth and function of most plants at very low micromolar concentrations.^3^ These effects substantially reduce crop yields, particularly when combined with other stresses, such as drought and nutrient deficiency. Al toxicity is therefore recognized as the major factor limiting agricultural productivity on acid soils which occupy ∼30% of territorial land area and up to 50% of the potential arable lands worldwide, and is exceeded only by drought among abiotic limitations to crop production.^4,5^ Moreover, Al toxicity is an important contributor to forest decline,^6^ posing a real threat to the global ecological environment.

Decades of research have established the central role of the secretion of organic acid anions (including malate, citrate and oxalate) in Al resistance in the main crops.^3,7,8^ These anions chelate and restrict Al ions from entering the root apex, the primary site of Al toxicity.^2^ Genetically enhancing their biosynthesis or extrusion significantly increases crop Al resistance and growth on acid soils.^9,10^ In *Arabidopsis*, *Al-ACTIVATED MALATE TRANSPORTER 1* (*AtALMT1*, the major contributor of Al resistance in *Arabidopsis*) and *MULTI-DRUG and TOXIC COMPOUND EXTRUSION* (*MATE*) respectively encode malate and citrate efflux channels/transporters conferring its resistance to Al toxicity.^11,12^ The Al-induced expression of both genes is exclusively controlled by the zinc finger transcription factor SENSITIVE TO PROTON TOXICITY 1 (STOP1), the central regulator of Al resistance.^12,13^ STOP1 has widespread conservation of function in Al resistance in different plant species.^14,15^ Whilst *STOP1* mRNA abundance is largely unresponsive to Al,^13^ Al promotes STOP1 protein accumulation in root cell nuclei, and its accumulation is regulated by the F-box protein REGULATION OF ATALMT1 EXPRESSION 1 (RAE1), which targets STOP1 degradation via the ubiquitin-26S proteasome pathway.^16^ Additionally, STOP1 is also regulated by SUMOylation and phosphorylation.^17,18^ Nevertheless, how Al ions are perceived and then connected to the accumulation of the STOP1 central regulator remains unknown.

Plant receptor-like kinases (RLKs), function as cell surface receptors for steroid hormone, chemical or peptide signals.^19–23^ We hence wondered whether RLKs might similarly serve in Al perception and/or signaling. A typical RLK consists of a ligand-binding extracellular domain, a single transmembrane domain, and a cytoplasmic serine/threonine kinase domain.^24^ The *Arabidopsis* genome contains > 600 genes encoding RLK,^24^ most of which have not been functionally characterized. In this study, we identified a novel Al resistance gene (here named *Al Resistance 1, ALR1*) encoding a typical leucine-rich-repeat (LRR) RLK, which was previously known as the phytosulfokine (PSK) peptide receptor kinase (PSKR1) involved in multiple processes,^25^ such as root growth and biotic stress response.^26–28^ We show here that this kinase is unexpectedly an Al ion receptor, and that the Al sensing conferred is physically and functionally separate from PSK sensing. In essence, our work defines a unique plant Al ion receptor ALR1, and reveals how its perception of Al ions is linked via NADPH oxidase RbohD, reactive oxygen species (ROS), RAE1 and STOP1 to the promotion of organic acid anion extrusion-dependent Al resistance.

## Results

### *ALR1* confers Al resistance

**Fig. 1:**
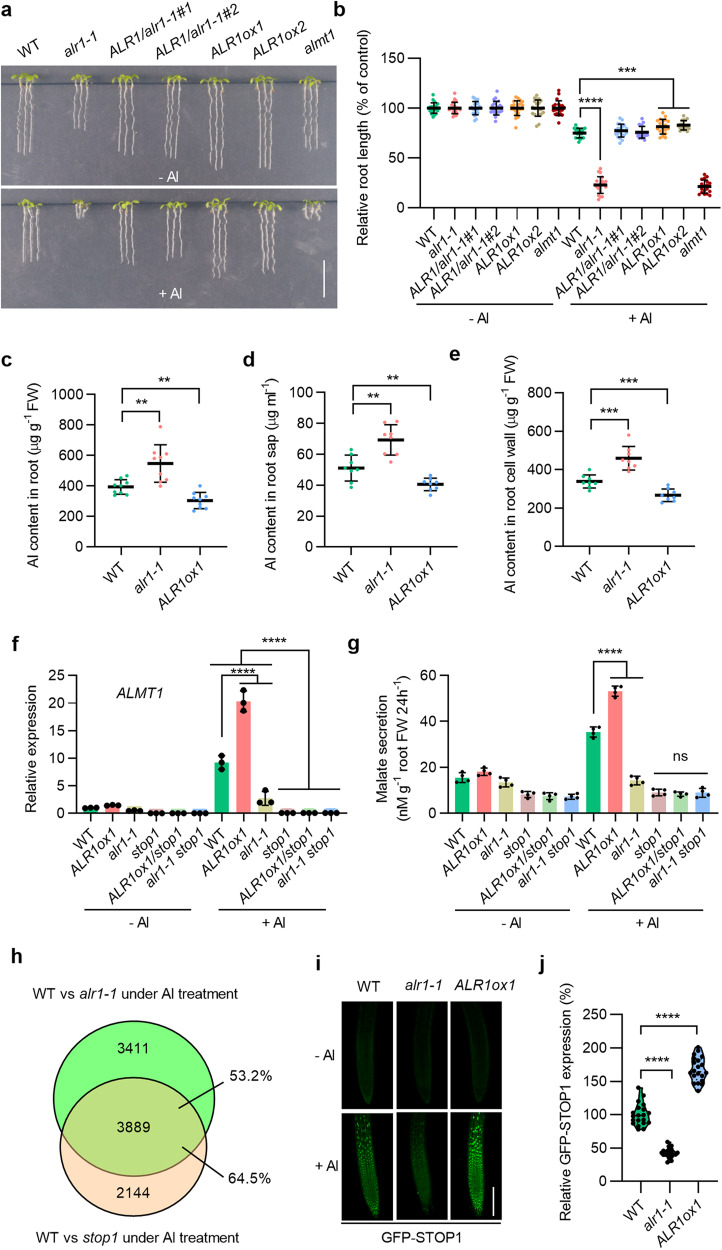
*ALR1* confers Al resistance. **a**, **b** Root growth under control and Al (1 mM) treatments (**a**), and their relative quantification (**b**) (*n* = 17–21). The average length of each genotype was set to 100%, and the relative root length was expressed as a percentage (root length with Al treatment/root length without Al × 100). *almt1* was used as a positive control. **c**–**e** Al content in 1 cm root tips (**c**), root sap (**d**) and root cell walls (**e**) under Al (25 µM) treatment (*n* = 9 in **c,** *n* = 8 in **d**, **e**). **f** Expression of *ALMT1* in roots under control and Al (25 µM) treatments (*n* = 3). **g** Malate secretion from roots under control and Al (50 µM) treatments (*n* = 4). **h** Venn diagram showing the overlap of differentially expressed genes (foldchange > 1.5) between WT vs *alr1-1* and WT vs *stop1* under Al treatment. **i**, **j** GFP-STOP1 fluorescence signals in roots (**i**) and their relative quantification under Al treatment (**j**) (*n* = 20). Bars = 1 cm (**a**), 100 µm (**i**). All data were analyzed by unpaired *t*-test (**b**–**f**, **g**, **j**), or two-way ANOVA (**f**) (ns non-significance, **P < 0.01, ***P < 0.001, ****P < 0.0001).

Screening a library of *RLK* T-DNA insertion mutants, we identified SALK_008585 (*alr1-1*) displaying remarkably reduced Al resistance (Fig. 1a, b). Whilst ALR1 is known to be required for root growth,^28^ the morphological development of *alr1-1* appears largely identical to that of wild type (WT) (Supplementary information, Fig. S1a). Despite the relatively shorter roots of *alr1-1* (vs WT) in control conditions, Al-mediated root growth inhibition is substantially greater in *alr1-1* and *alr1-2* (SALK_071659C) than in WT (Fig. 1a, b; Supplementary information, Fig. S1b, c). The reduced Al resistance of *alr1-1* was restored to normal by transgenic expression of *ALR1* (driven by the native *ALR1p* promoter; *ALR1/alr1-1#1* and *#2*), and Al resistance was promoted by overexpression of *ALR1* (*ALR1ox1* and *ox2*; Fig. 1a, b; Supplementary information, Fig. S1d). This increased Al sensitivity of *alr1-1* is not attributed to its deficit in root development, as other root-deficient mutants showed WT-like Al response (Supplementary information, Fig. S1e, f). The reduced Al resistance of *alr1-1* was next confirmed in hydroponic and soil conditions (Supplementary information, Fig. S1g–k). Further analyses indicated that *ALR1* is expressed in root and shoot, and that *ALR1* mRNA level (in WT plants) is relatively unaffected by low pH or Al treatments (Supplementary information, Fig. S2a, b). Additionally, the lack of ALR1 did not significantly affect plant responses to other metal ions (Supplementary information, Fig. S2c, d). We conclude that *ALR1* confers ion-specific resistance to Al stress.

We next found both root and shoot Al contents of Al-treated *alr1-1* seedlings were significantly increased vs WT, so were the symplastic and apoplastic Al contents in root, whilst those of *ALR1ox1* seedlings were significantly decreased (Fig. 1c–e; Supplementary information, Fig. S2e), suggesting that *ALR1* confers Al resistance via promoting Al exclusion. These effects of ALR1 are Al-specific, because the accumulation of other ions is largely unaffected in roots lacking ALR1 (Supplementary information, Fig. S2f). ALMT1- and MATE-mediated organic acid anion (malate and citrate) secretion is pivotal for *Arabidopsis* Al exclusion and resistance,^11,12^ and we accordingly found that ALMT1- and MATE-encoding mRNA abundances are reduced in *alr1-1* roots, but increased in *ALR1ox1* roots compared to WT upon Al treatment (Fig. 1f; Supplementary information, Fig. S3a). In consequence, malate and citrate secretion were reduced in *alr1-1*, but increased in *ALR1ox1* vs WT upon Al treatment (Fig. 1g; Supplementary information, Fig. S3b). We conclude that *ALR1* confers Al resistance by promoting the exclusion of Al from roots via the enhancement of Al-induced organic acid anion secretion.

### ALR1-promoted Al resistance is STOP1-dependent

Al-induced *ALMT1* and *MATE* expression is controlled by STOP1, the central transcriptional regulator of Al resistance.^12,13^ We next found *ALR1* and *STOP1* expression to be coordinated (Supplementary information, Fig. S3c), and that the enhanced Al resistance of *ALR1ox1* was completely suppressed by a *stop1* knockout mutant (Supplementary information, Fig. S3d, e). Moreover, an *alr1-1 stop1* double mutant displayed an Al sensitivity comparable to that of both single mutants (Supplementary information, Fig. S3f, g). Further analyses revealed that the promotion of Al-induced expression of *ALMT1* and *MATE* by ALR1 is dependent on STOP1, as is the promotion of Al-induced secretion of malate and citrate (Fig. 1f, g; Supplementary information, Fig. S3a, b). An additional transcriptome profiling showed that the expression of 64.5% genes regulated by STOP1 was likewise regulated by ALR1 under Al treatment (Fig. 1h). We conclude that ALR1-promoted Al resistance is STOP1-dependent.

Although *STOP1* mRNA levels were comparable in WT and *alr1-1* (Supplementary information, Fig. S3h), we found that Al-induction of GFP-STOP1 accumulation in root apex nuclei was greatly reduced in *alr1-1* (vs WT), but promoted by *ALR1* overexpression (Fig. 1i, j; Supplementary information, Fig. S3i), by using a *STOP1p:GFP-STOP1* transgenic line.

However, unlike *stop1* which is insensitive to phosphate (Pi) starvation in root growth,^29^ *alr1-1* showed a WT-like response (Supplementary information, Fig. S2g, h), indicating that ALR1 is not involved in response to Pi starvation. Furthermore, since the ALR1 orthologue PSKR2 functions redundantly with ALR1 in PSK signaling,^30^ we detected a relatively minor (relative to that of ALR1) contribution of PSKR2 to Al resistance (Supplementary information, Fig. S3j, k). Nevertheless, as in *alr1-1*, Al-induced STOP1 accumulation was not completely abolished in an *alr1-1 pskr2* double mutant (Supplementary information, Fig. S3l, m), perhaps suggesting additional functional redundancy or the existence of minor ALR1-independent mechanisms via which Al promotes STOP1 accumulation. We conclude that ALR1 is predominantly required for the Al-induced accumulation of STOP1.

### RbohD-dependent ROS promote STOP1 accumulation

**Fig. 2:**
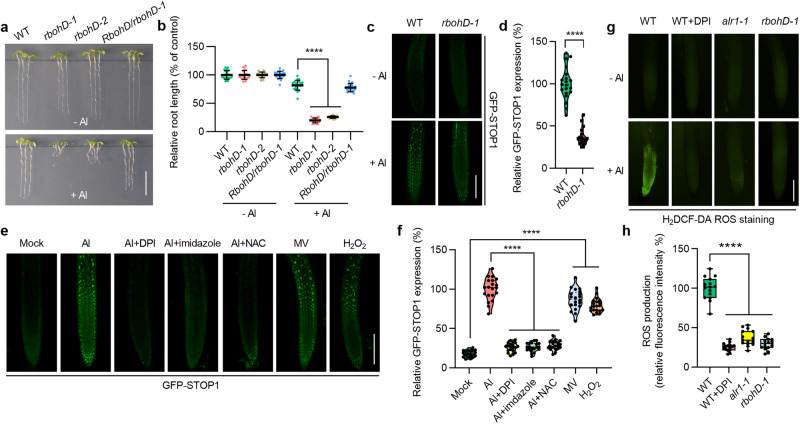
RbohD-dependent ROS promote STOP1 accumulation. **a**, **b** Root growth under control and Al (1 mM) treatments (**a**), and their relative quantification (**b**) (*n* = 20). The average length of each genotype was set to 100%, and the relative root length was expressed as percentage (root length with Al treatment/root length without Al × 100). **c**–**f** GFP-STOP1 fluorescence signals in roots (**c**, **e**) and their relative quantification under indicated treatments (**d**, **f**) (*n* = 20). **g**, **h** ROS visual signals in roots detected by H_2_DCF-DA (**g**), and their relative quantification under 10 min Al treatment (**h**) (*n* = 20). Bars = 1 cm (**a**), 100 µm (**c**, **e**, **g**). Data were analyzed by unpaired *t*-test (**b**, **d**, **f**, **h**) (ns non-significance, ****P < 0.0001).

With ALR1 failing to interact with STOP1 (Supplementary information, Fig. S4a), upon screening of ALR1 interactants in a split-ubiquitin membrane yeast two-hybrid system, we identified 18 potential interactors (Supplementary information, Table S1). Testing mutants lacking these potential interactors for Al sensitivity, we found that loss of RbohD function (*rbohD-1* and *rbohD-2*) showed substantially reduced Al resistance, a phenotype rescued by transgenic complementation with *RbohD* (Fig. 2a, b), suggesting that RbohD is a likely ALR1 substrate. We then confirmed the ALR1–RbohD interaction in yeast and planta (Supplementary information, Fig. S4b–d). Further experiments with additional root-expressed Rboh family members detected relatively weak ALR1–RbohA and ALR1–RbohE interactions (Supplementary information, Fig. S4e), and almost no effect on Al sensitivity (data not shown). Furthermore, the Al-induced accumulation of STOP1 was substantially compromised in the *rbohD* mutant (Fig. 2c, d; Supplementary information, Fig. S4f), as was Al-induced expression of *ALMT1* and *MATE* (Supplementary information, Fig. S4g, h), resulting in decreased Al-induced malate secretion (Supplementary information, Fig. S4i). These results in aggregate indicate that RbohD promotes Al resistance by enhancing the Al-induced accumulation of STOP1.

Because RbohD is an NADPH oxidase responsible for ROS generation,^31^ we next determined whether ROS promotes Al-induced STOP1 accumulation, finding that the NADPH oxidase inhibitors diphenyleneiodonium chloride (DPI) and imidazole and the ROS scavenger N-acetyl-L-cysteine (NAC) all suppressed Al-induced STOP1 accumulation (Fig. 2e, f; Supplementary information, Fig. S4j). In contrast, methylviologen (MV), which stimulates intracellular ROS generation, and hydrogen peroxide (H_2_O_2_) both induced STOP1 accumulation in the absence of Al (Fig. 2e, f; Supplementary information, Fig. S4j). In addition, *ALR1ox*-promoted STOP1 accumulation was substantially suppressed by DPI and NAC, whilst MV and H_2_O_2_ largely restored STOP1 accumulation in *alr1-1* and *rbohD* mutants (Supplementary information, Fig. S4k, l). Accordingly, H_2_O_2_ and MV induced the expression of *ALMT1* and *MATE* in the absence of Al (Supplementary information, Fig. S4m, n). Furthermore, we found ROS levels to be significantly increased 10 min after onset of Al treatment in WT root apices, but not in roots of plants lacking ALR1 or RbohD (Fig. 2g, h). We conclude that RbohD-dependent ROS production is necessary for Al-induced STOP1 accumulation, and thus for Al resistance.

### ALR1 phosphorylates RbohD to boost ROS production

Because ALR1 can interact with RbohD (Fig. S4b–d), we next tested if ALR1 could phosphorylate RbohD. We found that the recombinant ALR1 cytoplasmic domain (fused with a trigger factor tag, TF-ALR1^CD^; the TF tag is a 48 kDa chaperone that helps to decrease protein misfolding; Supplementary information, Fig. S5a) indeed phosphorylated an N-terminal fragment of RbohD (TF-RbohD^N^) in vitro (Supplementary information, Fig. S5a, b). Although initial mass spectrum analyses failed to identify the RbohD phosphorylation site, we subsequently found that a small region of RbohD^N^ had been inadvertently omitted from these analyses (Supplementary information, Fig. S5c). This region contains Ser39 (S39), a phosphosite that plays a key role in RbohD activation during the immune reaction.^32^ Focusing therefore on S39, we found that a S39 to Ala substitution (RbohD^N-S39A^) substantially reduced ALR1^CD^-mediated phosphorylation of RbohD^N^ (Supplementary information, Fig. S5b), indicating S39 to be a major potential target for ALR1-dependent RbohD phosphorylation. We next employed previously described pS39 antibodies specially recognizing the phospho-S39 form of RbohD,^32^ finding that RbohD^N^, but not RbohD^N-S39A^, was in vitro phosphorylated by ALR1^CD^ (Supplementary information, Fig. S5d).

**Fig. 3:**
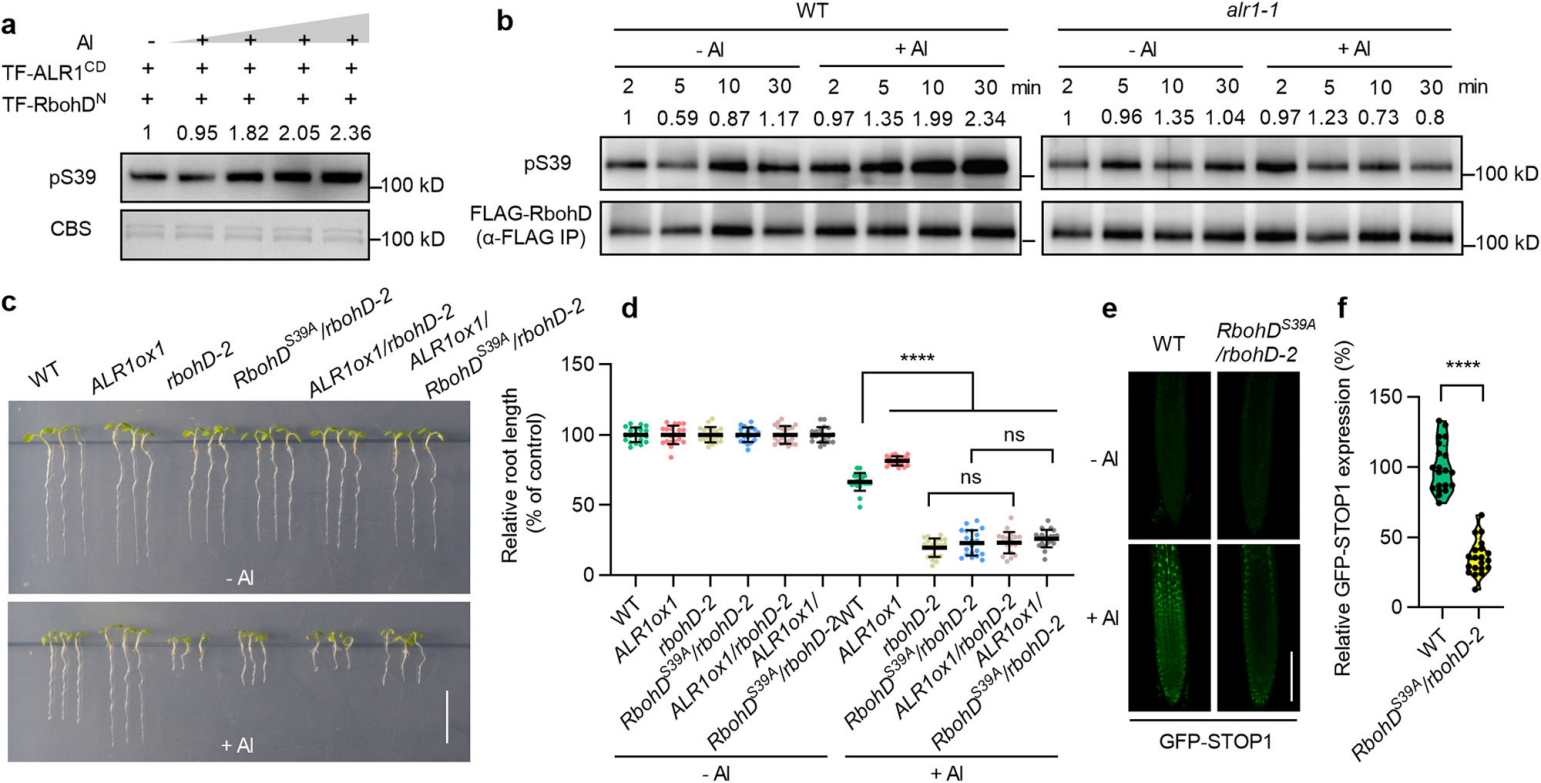
ALR1 phosphorylates RbohD boosting STOP1 accumulation. **a** in vitro phosphorylation of RbohD in response to different concentrations of Al (0, 1, 10, 20 and 100 nM) detected with pS39 antibodies. CBS indicates coomassie blue staining. **b** in vivo phosphorylation of RbohD in seedlings following control or Al (50 µM) treatments. **c**, **d** Root growth under control and Al (1 mM) treatments (**c**), and their relative quantification (**d**) (*n* = 20). The average length of each genotype was set to 100%, and the relative root length was expressed as a percentage (root length with Al treatment/root length without Al × 100). **e**, **f** GFP-STOP1 fluorescence signals in roots (**e**) and their relative quantification under Al treatment (**f**) (*n* = 20). Bars = 1 cm (**c**), 100 µm (**e**). Data were analyzed by unpaired *t*-test (**d**, **f**) (ns non-significance, ****P < 0.0001).

Further analyses revealed that Al treatment promotes ALR1^CD^-mediated phosphorylation of RbohD S39, and that this promotion increases with increasing Al concentration (Fig. 3a). This effect is relatively specific to Al, because other metal ions, in particular lanthanum (La^3+^; an ion structurally similar to Al^3+^), do not promote ALR1-dependent RbohD S39 phosphorylation (Supplementary information, Fig. S5e, f). Finally, phosphorylation was abolished when RbohD^N^ was incubated with a kinase-dead variant ALR1^CD^ (ALR1^CD-K762R^; Supplementary information, Fig. S5g). These observations suggest that the ALR1 cytoplasmic domain, likely mainly the kinase domain, specifically senses Al ion concentration and proportionately phosphorylates RbohD in response.

We next found that Al enhances RbohD S39 phosphorylation in WT, but not in *alr1-1* (Fig. 3b), indicating that ALR1 is required for in vivo Al-activated RbohD S39 phosphorylation. In addition, transgenic expression of mutant *RbohD*^*S39A*^ did not restore Al resistance in *rbohD* plants (vs WT; Fig. 3c, d), whilst lack of RbohD or expression of *RbohD*^*S39A*^ suppressed Al resistance conferred by overexpression of *ALR1* (Fig. 3c, d). In contrast, plants expressing a constitutive RbohD phosphorylation mimic (RbohD^S39D^) exhibited increased Al resistance in the *rbohD* background vs WT, and rescued Al sensitivity of *alr1-1* (Supplementary information, Fig. S6a–d). Furthermore, Al-induced STOP1 accumulation and malate secretion was reduced by *RbohD*^*S39A*^ expression (Fig. 3e, f; Supplementary information, Figs. S4i, 5h), but increased by *RbohD*^*S39D*^ expression (Supplementary information, Fig. S6e–g). These observations confirm that *RbohD* is epistatic to *ALR1*, and that Al-induced ALR1-mediated phosphorylation of RbohD S39 promotes STOP1 accumulation, thus conferring Al resistance.

Further analyses revealed that Al treatment stimulated NADPH oxidase activity in WT plants, but not in *alr1-1* or *rbohD* mutants, or *RbohD*^*S39A*^-expressing plants (Supplementary information, Fig. S5i). Accordingly, Al-promoted ROS generation was abolished in *rbohD* and *RbohD*^*S39A*^-expressing plants (vs WT; Supplementary information, Fig. S5j, k), but increased in *RbohD*^*S39D*^-expressing plants (Supplementary information, Fig. S6h, i). We conclude that ALR1 confers Al resistance via Al-dependent phosphorylation of RbohD, which in turn stimulates RbohD-dependent ROS generation and associated STOP1 accumulation.

### ALR1 function requires SERK co-receptors

**Fig. 4:**
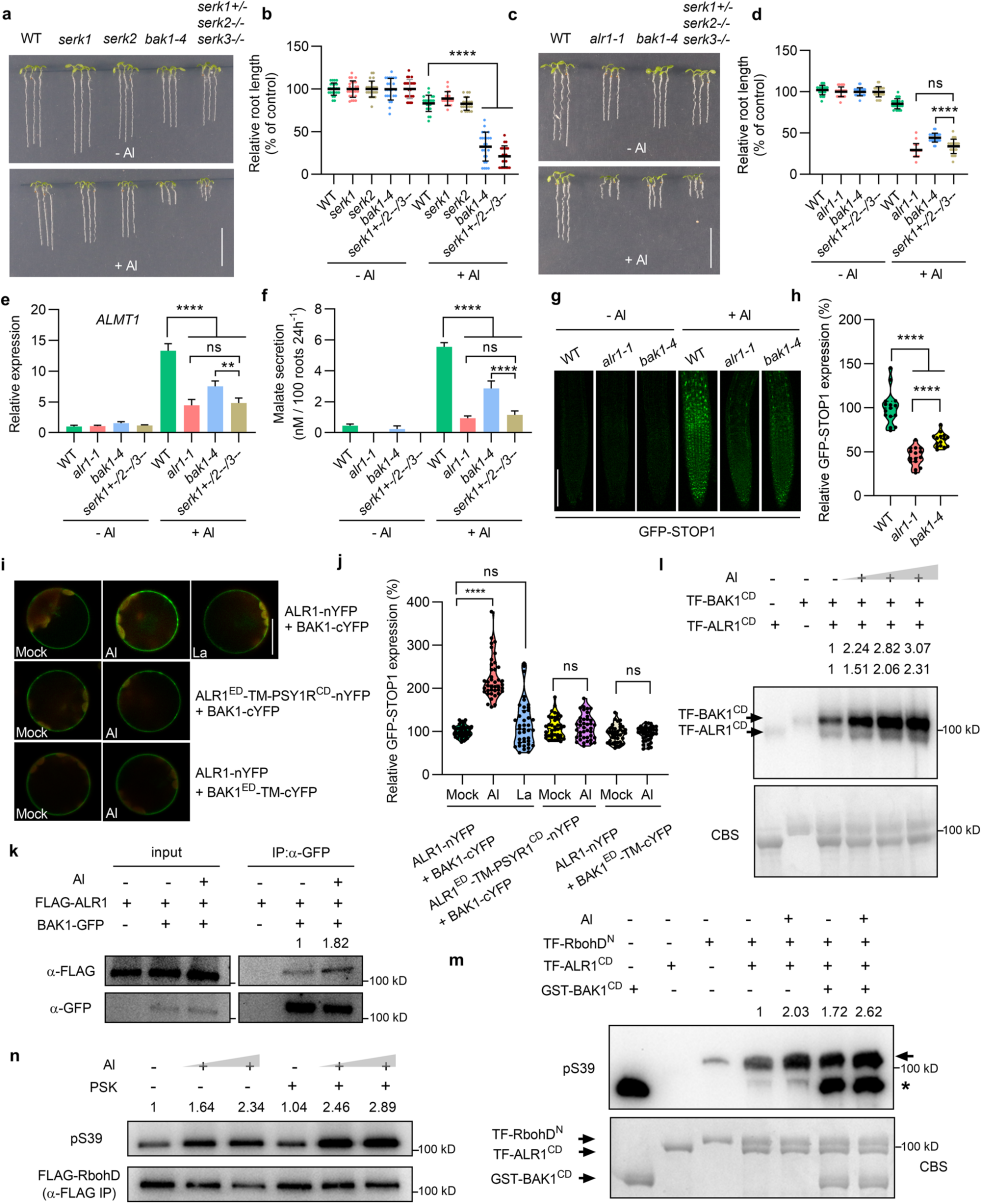
BAK1 contributes to ALR1-mediated Al resistance and signaling. **a**–**d** Root growth under control and Al treatments (**a**, **c**), and their relative quantification (**b**, **d**) (*n* = 19–24). The average length of each genotype was set to 100%, and the relative root length was expressed as a percentage (root length with Al treatment/root length without Al × 100). **e** Expression of *ALMT1* in roots under control and Al (25 µM) treatments for 6 h (*n* = 3). **f** Malate secretion from roots under control and Al (25 µM) treatments for 24 h (*n* = 3). **g**, **h** GFP-STOP1 fluorescence signals in roots (**g**) and their relative quantification (**h**) (*n* = 15). **i** Bimolecular fluorescence complementation (BiFC) mediated detection of interaction between ALR1 and BAK1, ALR1^ED^-TM-PSYR1^CD^ (a chimeric ALR1 comprised of ALR1 extracellular and transmembrane domains and the cytoplasmic domain of another RLK PSY1R) and BAK1, or ALR1 and BAK1^ED^-TM (BAK1 extracellular domain and transmembrane domain), respectively, following control, Al (50 µM) or La (50 µM) treatments for 10 min. **j** Relative fluorescence intensity in **i** (*n* = 40). **k** Co-immunoprecipitation (Co-IP) showing the interaction between ALR1 and BAK1 in protoplasts following control or Al (50 µM) treatments for 10 min. **l** Inter-phosphorylation of ALR1^CD^ and BAK1^CD^ in response to different concentrations of Al (0, 10, 50 and 100 nM) in vitro. **m** BAK1^CD^ promotes the phosphorylation of RbohD^N^ by ALR1^CD^ under both control and 100 nM Al treatment conditions. The arrow indicates a phosphorylated RbohD^N^ band detected with pS39 antibodies. The asterisk indicates that pS39 antibodies can also unexpectedly recognize the BAK1 cytoplasmic domain (but cannot recognize other cytoplasmic domains, data not shown). **n** The effect of PSK (3 µM) on in vivo RbohD phosphorylation. Bars = 1 cm (**a**, **c**), 100 µm (**g**), 20 µm (**i**). All data were analyzed by unpaired *t*-test (**b**, **d**–**f**, **h**, **j**) (ns non-significance, *P < 0.05, **P < 0.01, ****P < 0.0001).

Because the interaction of ALR1 with somatic embryogenesis receptor-like kinase (SERK) co-receptors is required for PSK signaling,^28^ we next found that lack of SERK3/BAK1 (*serk3*/*bak1-4* mutant), but not of SERK1 or SERK2, obviously reduced Al resistance vs WT, although this reduction was less than that conferred by *serk1*^*+/*–^ *serk2*^–*/*–^ *serk3*^–*/*–^ triple mutant (Fig. 4a, b). Moreover, the Al resistance and Al-induced *ALMT1* expression and malate secretion were all reduced in *bak1-4* and *serk1*^*+/*–^ *serk2*^–*/*–^ *serk3*^–*/*–^ triple mutants vs WT, and the reduced levels in the triple mutant were comparable to those in *alr1-1* mutant (Fig. 4c–f). With BAK1 being a major SERK in Al response, we next found that the Al-induced STOP1 accumulation was substantially reduced in *bak1-4* vs WT, although the reduction was less than in *alr1-1* (Fig. 4g, h). Further experiments demonstrated that Al promotes the interaction between ALR1 and BAK1, and does so in an ALR1 cytoplasmic domain-dependent manner (Fig. 4i–k), thus boosting ALR1-BAK1 inter-phosphorylation (Fig. 4l). Additionally, BAK1, whilst unable to itself phosphorylate RbohD, facilitates Al-promoted ALR1-dependent phosphorylation of RbohD (Fig. 4m). In accord with the previous demonstration that the PSK peptide promotes in vivo ALR1–BAK1 interactions,^28^ we also found that PSK application increases Al-dependent RbohD phosphorylation (Fig. 4n). We conclude that the ALR1–BAK1 interaction is required for a complete Al-promoted ALR1-dependent RbohD phosphorylation, and is thus involved in Al resistance.

### ROS modify RAE1 to facilitate STOP1 accumulation

Because STOP1 proteolysis is mediated by the RAE1 F-box protein,^16^ we next determined whether ROS affect this process. By ectopically expressing STOP1-GFP in mesophyll protoplasts (which do not natively express STOP1, even in response to Al), we found that whilst co-expression of RAE1 reduced STOP1-GFP accumulation, this reduction was suppressed by MV, with MV-induced suppression being in turn inhibited by NAC (Supplementary information, Fig. S7a, b). These observations suggest that ROS directly inhibits RAE1-mediated STOP1 proteolysis.

ROS signaling is often mediated via post-translational modification of cysteine (Cys) residues with low p*K*_a_.^33^ Biotin-conjugated iodoacetamide (BIAM) specifically competes with ROS in reacting with target Cys residues, enabling ROS-sensitive Cys residues to be detected by BIAM labeling.^34^ We found weakly BIAM-modified recombinant STOP1 in vitro (Supplementary information, Fig. S7c). Although subsequent mass spectrum analyses revealed 3 major potential ROS-modified STOP1 Cys residues (Cys27, Cys185 and Cys335), mutational substitution of these Cys residues with Ala residues did not detectably affect either RAE1-mediated STOP1 degradation or ROS-mediated suppression of STOP1 proteolysis (Supplementary information, Fig. S7d), implying that ROS-mediated inhibition of RAE1-dependent STOP1 proteolysis is unlikely to be dependent upon post-translational modification of STOP1.

**Fig. 5:**
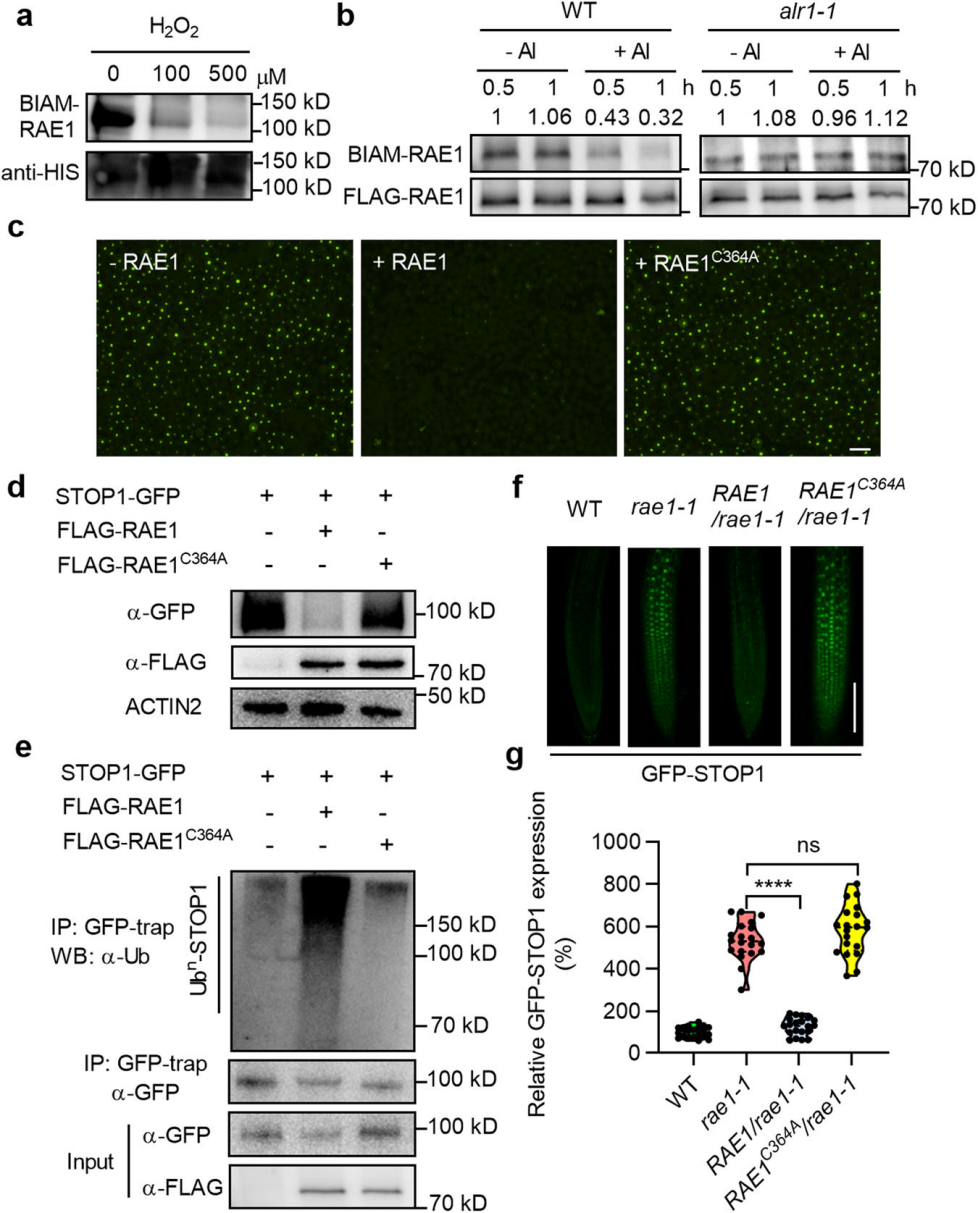
ROS modify RAE1 to facilitate STOP1 accumulation. **a** in vitro labeling of BIAM for recombinant His-RAE1. **b** in vivo labeling of BIAM for FLAG-RAE1 immuno-precipitated from seedlings following 20 min control and Al (50 µM) treatments. **c** STOP1-GFP expression in mesophyll protoplasts with or without co-expressing RAE1 or mutated RAE1 (RAE1^C364A^). **d** Abundance of STOP1-GFP in protoplasts detected by α-GFP antibody. **e** Ubiquitination of STOP1-GFP in protoplasts detected by α-Ub antibody. **f**, **g** GFP-STOP1 fluorescence signals in roots (**f**) and their relative quantification under control condition (**g**) (*n* = 20). Bars = 100 µm (**c**, **f**). Data were analyzed by unpaired *t*-test (ns non-significance, ****P < 0.0001).

We next identified strong H_2_O_2_-competable modification of recombinant RAE1 in vitro (Fig. 5a), and found that BIAM labeling of in vivo FLAG-RAE1 was suppressed in WT by Al treatment, but not in *alr1-1* (Fig. 5b), suggesting that Al promotes the oxidative modification of RAE1 in an ALR1-dependent manner. Mass spectrum analyses further detected 9 BIAM-modified RAE1 Cys residues (Supplementary information, Fig. S7e, f), among which Cys364 is predominantly required for RAE1-promoted STOP1 degradation (Fig. 5c, d; Supplementary information, Fig. S7g, h). Accordingly, STOP1 ubiquitination was substantially reduced by RAE1^C364A^ co-expression (vs WT RAE1 co-expression; Fig. 5e). Intriguingly, the Cys364-to-Ala364 substitution did not detectably affect the in vivo interaction between RAE1 and STOP1 (Supplementary information, Fig. S7i, j), indicating that Cys364, although necessary for RAE1 function, is not necessary for RAE1–STOP1 interaction. Confirming the role of Cys364 in RAE1 function in vivo, we found that transgenic expression of *RAE1*^*C364A*^ in a *rae1-1* loss-of-function mutant (*RAE1*^*C364A*^/*rae1-1*) enabled STOP1 accumulation in the absence of Al to a level similar to that in the *rae1-1* control, whilst STOP1 accumulation was not detected in *RAE1/rae1-1* (Fig. 5f, g; Supplementary information, Fig. S7k). Overall, these results demonstrated that Al-induced ALR1-promoted ROS generation enhances STOP1 accumulation via oxidative modification of RAE1 Cys364.

### ALR1 is an Al ion receptor

**Fig. 6:**
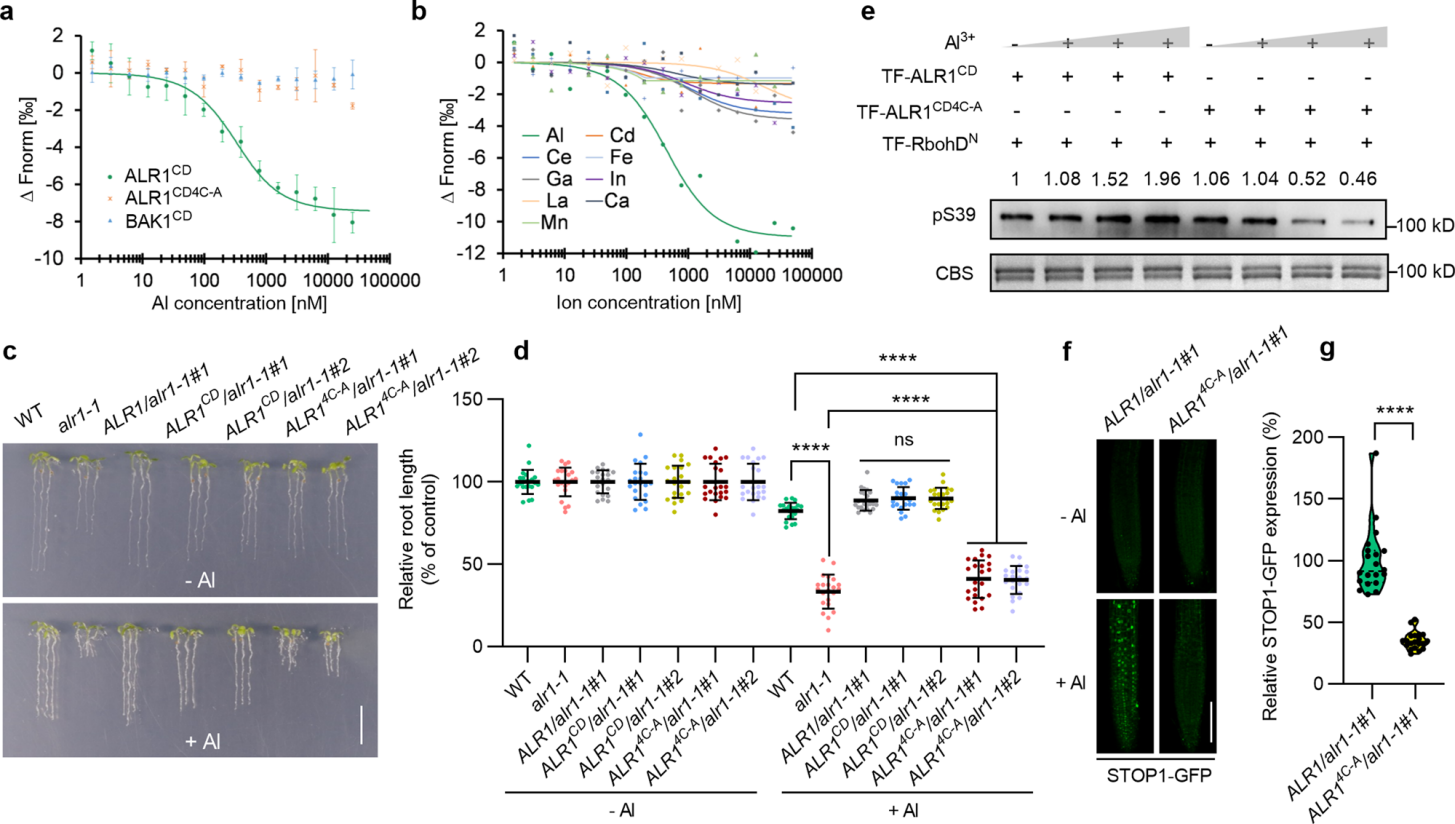
ALR1 is an Al ion receptor. **a** Quantification of the binding affinity of ALR1^CD^, mutated ALR1^CD^ (ALR1^CD4C-A^) and BAK1^CD^ for Al ions by MST (*n* = 4 for ALR1^CD^, *n* = 3 for ALR1^CD4C-A^ and BAK1^CD^). **b** Quantification of the binding affinity of ALR1^CD^ for metal ions other than Al by MST. **c**, **d** Root growth under control and Al treatment (**c**), and their relative quantification (**d**) (*n* = 20). The average length of each genotype was set to 100%, and the relative root length was expressed as a percentage (root length with Al treatment/root length without Al × 100). **e** Phosphorylation of RbohD^N^ by ALR1^CD^ or ALR1^CD4C-A^ in response to Al. **f**, **g** GFP-STOP1 fluorescence signals in roots (**f**) and their relative quantification under Al treatment (**g**) (*n* = 20). Bars = 1 cm (**c**), 100 µm (**f**). All data were analyzed by unpaired *t*-test (**d**, **g**) (ns non-significance, ****P < 0.0001).

Because ALR1^CD^-mediated phosphorylation of RbohD is responsive to Al concentration (Fig. 3a, b), we next determined if ALR1 binds Al ions specifically. Using a microscale thermophoresis (MST) assay to detect binding to a recombinant ALR1^CD^ protein, we found that Al ions display a high binding affinity for ALR1^CD^ (Fig. 6a; dissociation constant ranges from 0.2 µM to 2 µM upon different batches of experiments). This binding was confirmed via isothermal titration calorimetry (Supplementary information, Fig. S8a–e). The binding affinity may partially rely on its kinase activity, as a kinase-dead mutation (ALR1^CD-K762R^) increases the dissociation constant by 5–10 fold (Supplementary information, Fig. S8f). Moreover, ALR1^CD^ did not detectably bind other metal ions (Fig. 6b), suggesting that ionic binding to ALR1^CD^ is Al-specific. We additionally showed that the BAK1 cytoplasmic domain does not bind Al ions (Fig. 6a), indicating that BAK1 is not directly involved in Al perception. Furthermore, a truncated ALR1 (spanning ALR1 residues 658 to 1008) lacking the extracellular domain is sufficient to restore Al resistance in *alr1-1* knockout mutant (*ALR1*^*CD*^*/alr1-1*; Fig. 6c, d), favoring that the intracellular ALR1 cytoplasmic domain is responsible for Al perception.

Since many amino acid residues (e.g., Asp, Glu, Asn, Cys, etc.) can be the candidates for metal ion targets,^35^ we focused initially on the least distributed Cys residues in the ALR1 cytoplasmic domain, and determined if the 8 Cys residues contribute to Al perception. Mutational analyses of these Cys groups (but not of Cys797, where mutation failed) revealed that substitution of Cys939/944 or Cys985/987 with Ala, affected the Al-binding of ALR1^CD^ (Supplementary information, Fig. S8g–l), and that a simultaneous quadruple Ala substitution (Cys-939/944/985/987-Ala; ALR1^CD4C-A^) completely abolished the binding (Fig. 6a), indicating that the 4 Cys residues are necessary for ALR1 to recognize Al ions.

The above identified 4 Cys residues (Cys-939/944/985/987) are located in a C-terminal region of the ALR1 cytoplasmic domain (Supplementary information, Fig. S8h). To exclude the possibility that mutation of these Cys residues affects the basal kinase activity or overall structural integrity of ALR1^CD^, we next found the auto-phosphorylation of recombinant ALR1^CD^ and ALR1^CD4C-A^ to be comparable, as is the phosphorylation of RbohD and BAK1 by ALR1^CD^ and ALR1^CD4C-A^ (Supplementary information, Fig. S9a–c). Additionally, transgenic expression from the native *ALR1* promoter of mutant *ALR1*^*4C-A*^ (encoding a full-length ALR1 with Cys939/944/985/987 all substituted by Ala) in *alr1-1* (*ALR1*^*4C-A*^*/alr1-1*) conferred a normal PSK response (as compared with *ALR1/alr1-1#1* and *alr1-1*), whilst the kinase-dead control (*ALR1*^*K762R*^*/alr1-1*) displayed an abolished PSK response (Supplementary information, Fig. S9d, e). Finally, structural integrity determinations using circular dichroism (CD) revealed the structures of mutant (ALR1^CD4C-A^) and WT (ALR1^CD^) proteins to be almost identical (Supplementary information, Fig. S9f, g). These data demonstrate that substitution of these four Cys residues (Cys939/944/985/987) with Ala does not detectably affect the structural integrity or kinase activity of the ALR1 cytoplasmic domain.

We next found that Al-promoted phosphorylation of RbohD was largely abolished by ALR1^CD4C-A^, and that ALR1^CD4C-A^ kinase activity, unlike that of ALR1^CD^, was actually increasingly inhibited by increasing Al concentration (Fig. 6e), indicating that these 4 Cys residues are essential for Al-promoted ALR1 kinase activity, and that the disruption of Al binding from their substitution with Ala may cause ALR1^CD^ to be more vulnerable to toxic Al ions. Confirming the in vivo function of these 4 Cys residues, we next found that transgenic expression of the mutated *ALR1*^*4C-A*^ (*ALR1*^*4C-A*^*/alr1-1*) substantially reduced Al resistance (vs WT and *ALR1/alr1-1*; Fig. 6c, d). Accordingly, Al-activated root apex NADPH oxidase activity and ROS generation were both markedly decreased in *ALR1*^*4C-A*^*/alr1-1* lines (vs WT), but restored by *ALR1*^*CD*^ in *alr1-1* (Supplementary information, Fig. S10a–c). Furthermore, Al-induced STOP1 accumulation was substantially reduced in *ALR1*^*4C-A*^*/alr1-1* plants (vs WT; Fig. 6f, g; Supplementary information, Fig. S10d). These results collectively demonstrate that ALR1 is an Al ion receptor.

### ALR1-mediated Al perception is independent of its PSK sensing function

ALR1 senses PSK peptide through its extracellular domain.^28^ Our further studies revealed that mutation of the PSK peptide-binding sites in ALR1 (in *ALR1*^*F506A*^*/alr1-1*, *ALR1*^*R300A*^*/alr1-1* transgenic lines) does not obviously affect Al resistance, whilst a kinase-dead mutation (in *ALR1*^*K762R*^*/alr1-1*) does (Supplementary information, Fig. S11), indicating that ALR1-dependent Al perception is likely independent of its PSK sensing function.

Since binding of PSK or Al ion both enhances the interaction between ALR1 and SERK co-receptors, we indeed found that exogenous application of PSK peptide or overexpression of *PSK* genes increased Al resistance in an ALR1-dependent manner (Supplementary information, Fig. S12a–d), and that *PSK* overexpression promotes STOP1-dependent Al signaling (Supplementary information, Fig. S12e–h). Nevertheless, PSK cannot induce Al signaling (e.g., ROS production, STOP1 accumulation, *ALMT1* expression) in the absence of Al (Supplementary information, Figs. S12e–g, S13a, b), and PSK promotes root growth independently of RbohD (Supplementary information, Fig. S13c, d). These suggest that although they share same receptor and co-receptors, Al and PSK signaling pathways are separate.

In summary, we demonstrate that ALR1 is an Al ion receptor. Binding of Al ions to the ALR1 cytoplasmic domain promotes ALR1-mediated phosphorylation of RbohD, thus increasing ROS production. The consequent ROS-dependent accumulation of STOP1 stimulates organic acid anion secretion and results in enhanced Al resistance.

## Discussion

In this study, we report the unique discovery that the plant ALR1 receptor-like kinase is an Al ion receptor. Our discovery rests on the following evidence: (1) the cytoplasmic domain of ALR1 specifically binds Al ions; (2) the binding of Al to ALR1 recruits BAK1 co-receptor and promotes ALR1-mediated phosphorylation of RbohD, thus stimulating an Al-dependent elevation of ROS generation and signaling; (3) ALR1-dependent RbohD phosphorylation is quantitatively responsive to varying Al concentration and confers commensurate quantitative regulation of STOP1 accumulation; (4) abolition of the ALR1 Al binding function substantially suppresses the predominant STOP1-dependent Al signaling pathway.

Plants constantly sense and respond to a wide diversity of soil ions, including nutrient and toxic ions. Nevertheless, the mechanisms by which plants initially sense these different ions and transduce these signals remain poorly understood. Recent sensor/receptor discoveries have advanced understanding of how plants adaptively sense and respond to a variety of hormonal, chemical and physical cues, thus optimizing growth, survival and reproductive success,^21,36–38^ and the discovery of plant ion receptors will be similarly revealing. The finding that the plant ALR1 kinase is an Al ion receptor is unique because in mammals, ions are mostly sensed by ion channels. For example, mice sense sodium via the sodium ion (Na^+^) channel ENaC, and lack of ENaC in taste cells blocks mouse neural and behavioral Na^+^ responses.^39^ Furthermore, although the nitrate ion is at least partially sensed by the plant nitrate transporter NRT1.1,^40^ plant ion perception is more generally thought to be achieved via non-channel ion receptors. Accordingly, initial Na^+^ sensing by the sphingolipid GIPC subsequently activates *Arabidopsis* Ca^2+^ signaling,^41^ the intracellular Fe^3+^ is potentially sensed by HRZ/BTS RING ubiquitin ligases,^42^ and Zn^2+^ is likely to be sensed by plant bZIP transcription factors.^43^ Recent research has revealed that nitrate is also sensed by NLP7 transcription factor.^44^ So far as we are aware, our discovery that ALR1 is an Al ion receptor is unique because no kinase-type receptor has previously been shown to sense an inorganic or metal ion in all living organisms. Furthermore, in contrast to other RLKs, which perceive signals via their extracellular domains,^45^ ALR1 senses intracellular Al ions via its intracellular cytoplasmic domain, likely mainly the kinase domain. The binding of Al ions to the ALR1 cytoplasmic domain promotes ALR1-BAK1 association and inter-phosphorylation (Fig. 4i–l), suggesting a special mechanism for RLK activation. These findings thus provide novel insights into how living organisms perceive and respond to ions.

We have shown that a set of four C-terminal Cys residues (Cys939/944/985/987) are necessary for the Al binding function of ALR1 (Fig. 6). Nevertheless, our structural model suggests that they are unlikely to form an Al ion coordination center in space (Supplementary information, Fig. S8m), and their substitutions with Ala are more likely to indirectly affect Al binding. We propose that the ALR1 Al binding sites likely reside within the region spanned by Cys939 and Cys987. Although we have recently defined the Al binding sites of the ALMT1 protein via structural analysis,^46^ it is at present unclear if the ALR1 Al binding sites have a similar composition, and biochemical approaches are unlikely to pinpoint it. Future comparisons of the high-resolution structures of ALR1 (perhaps in complex with BAK1 and RbohD) in both Al-bound and unbound states will likely reveal how Al ions bind to ALR1 and how this binding promotes ALR1 kinase activity.

Our results also show that ALR1-mediated Al signaling (via its cytoplasmic domain) is functionally separate from ALR1-mediated PSK signaling (via its extracellular domain) (Fig. 6c, d; Supplementary information, Figs. S11c, d, S13a–d). However, the possibility, that these functionally separate Al and PSK signaling pathways interact, needs to be considered. For example, we here show that Al treatment represses the expression of most PSK-encoding genes (Supplementary information, Fig. S13E), implying that the PSK peptide is unlikely to explain Al resistance in nature. Because PSK signaling promotes root growth,^47^ the Al-induced repression of *PSK* expression may be an adaptive response to reduce the exposure of roots to toxic Al ions. Therefore, ALR1 may have dual functions concerning root growth under Al stress conditions, with the extracellular domain controlling normal root growth and the cytoplasmic domain regulating the Al stress response. In contrast, cytoplasmic kinase-domain-mediated Al signaling detoxifies Al and protects root tips from Al-induced cellular damages. Once Al toxicity is relieved, root growth recovers as PSK signaling is restored. ALR1 thus represents a so-far unique RLK that perceives extracellular and intracellular signals via distinct extracellular and intracellular protein domains to coordinate developmental and stress responses. Given the dual function of ALR1, we propose to name the gene *PSKR1/ALR1* (*Al Receptor 1*) for its future identity. Nevertheless, how PSKR1/ALR1 transduces PSK or Al signals to different downstream components remains an open question that needs to be addressed in future studies. Besides, since Al-induced STOP1 accumulation is not completely blocked in the *alr1-1* and *rbohD-1* mutants, additional ALR1-independent pathways may operate to fine-tune STOP1 expression.

Although ROS production in response to Al was previously thought to be Al toxicity syndrome,^1^ we give the direct evidence that the RbohD-dependent ROS are key second messengers in Al signaling linking membrane-based intracellular Al perception to nuclear response. we identify Cys364 of RAE1 is both oxidatively modified by ROS and necessary for RAE1 function and Al signaling (Fig. 5). Ala-substitution of Cys364 did not detectably affect the RAE1–STOP1 interaction (Supplementary information, Fig. S7i, j), and Ala-substitution assay of additional Cys residues indicated that they are less important for RAE1 function than Cys364, suggesting that Cys364 may function in the formation of an inter-molecular disulfide bond that facilitates the interaction between RAE1 and unknown components of the proteolytic machinery (e.g., the SCF E3 ubiquitin ligase complex). The finding that ROS directly act on the RAE1 F-box protein, the component of cellular proteolytic machinery, provides a novel ROS-signaling mechanism that has not been uncovered in plants.^48^ In addition, because MV or H_2_O_2_ promotes STOP1 accumulation in the absence of Al (Fig. 2e), any treatment that induces ROS production would likely more or less prevent STOP1 degradation and induce the expression of *ALMT1* and *MATE* genes. However, this would not lead to a large amount of malate and citrate release, as these transporters/channels still require extracellular Al^3+^ to trigger their activity.^11,46^

**Fig. 7:**
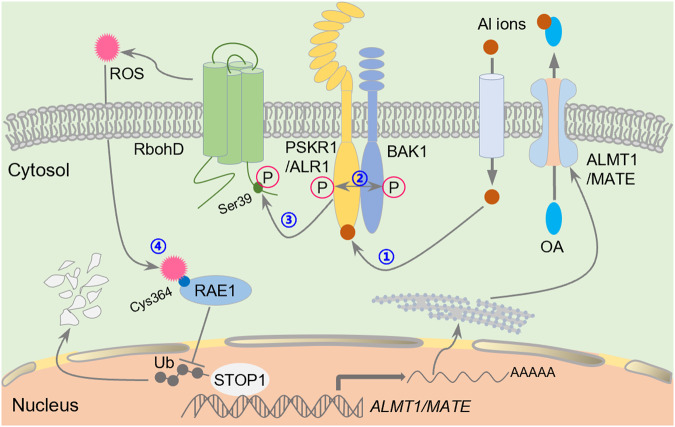
A summary model showing PSKR1/ALR1-dependent Al perception and signaling. ①Al ions entering root cells are perceived by the cytoplasmic domain of the PSKR1/ALR1 receptor. ②The binding of Al ions enables PSKR1/ALR1 to recruit BAK1, the representative of SERKs, as a co-receptor for inter-phosphorylation and activation, and thus ③promotes ALR1-mediated phosphorylation of the NADPH oxidase RbohD at Ser39, hence increasing ROS generation. ④The resultant accumulated ROS in turn inhibits the function of the RAE1 F-box protein via oxidative modification of Cys364 residue, thus suppressing RAE1-mediated degradation of STOP1, the master transcriptional regulator of Al-resistance genes. The consequent accumulation of STOP1 hence activates the expression of downstream genes, including the malate channel-encoding *ALMT1* gene, finally promoting organic acid anions secretion to chelate extracellular Al ions, thus conferring Al resistance. OA represents organic acid anions.

In conclusion, we have discovered ALR1, an LRR-RLK that is an Al ion receptor, and have revealed all subsequent steps in a ROS-mediated Al signaling pathway leading from ALR1 perception of Al ions to the STOP1 central regulator conferring Al resistance (Fig. 7). These discoveries will inform future molecular breeding of Al resistant crops and trees, and thus have huge potential not only for sustaining agricultural production and global food security, but also for forest restoration and improvement of the global ecological environment.

## Materials and methods

### Plant materials and growth conditions

*Arabidopsis thaliana* lines are in Col-0 genetic background. *alr1-1* (SALK_008585), *alr1-2* (SALK_071659C), *rbohD-1* (SALK_005253C) and *stop1* (SALK_114108) were obtained from *Arabidopsis* Biological Resource Center (ABRC). *rbohD-2*, *RbohD*^*S39A*^*/rbohD-2*, *rae1-1*, *serk1*, *serk2*, *serk3* (*bak1-4*), *serk1*^*+/*–^ *serk2*^–*/*–^ *serk3*^–*/*–^, and *STOP1p:GFP-STOP1/stop1* were described previously.^16,28,29,32,49^ *STOP1p:GFP-STOP1* lines in genetic background of *alr1-1*, *bak1-4*, *alr1-1/pskr2*, *ALR1ox1*, *rbohD-1*, *RbohD*^*S39A*^*/rbohD-2*, *RbohD*^*S39D*^*/rbohD-1* and *rae1-1*, were generated by crossing *STOP1p:GFP-STOP1/stop1* to *alr1-1*, *bak1-4*, *alr1-1/pskr2*, *ALR1ox1*, *rbohD-1*, *RbohD*^*S39A*^*/rbohD-2*, *RbohD*^*S39D*^*/rbohD-1* and *rae1-1*, respectively. The *short-root* (*shr*) and *rgi1 rgi2* mutants that are deficient in root growth were used as the negative controls. SHR is a transcription factor that regulates root radial patterning and stem cell niche maintenance,^50^ and RGI1/2 are receptor-like kinases responsible for RGF peptide perception in regulation of root stem cell niche.^51^ All the mutants were identified by PCR and confirmed by Sanger sequencing.

Plants used for treatment or propagation were grown in an environmental controlled growth chamber or room programmed for a 16-h-light/8-h-dark cycle with a daytime temperature of 23 °C and a night temperature of 21°C. For plants used for mesophyll protoplast isolation, the condition of a 10-h-light/14-h-dark cycle was conducted. The half-strength Murashige & Skoog (MS) medium was prepared for normal seedling growth as previously described.^52^

### Constructs and transgenic plants

For *ALR1* transgenic complementation test, the coding sequences of full-length *ALR1* (3080 bp) and truncated *ALR1* (ALR1^CD^, 1053 bp) driven by *ALR1* native promoter (3080 bp) were cloned into *pCambia1301* binary vector to generate *ALR1p:ALR1* and *ALR1p:ALR1*^*CD*^, respectively. The *ALR1p:ALR1*^*4C-A*^, *ALR1p:ALR1*^*R300A*^, *ALR1p:ALR1*^*F506A*^ and *ALR1p:ALR1*^*K762R*^ constructs were generated by site-directed mutagenesis in *ALR1p:ALR1* using the KOD-Plus-Mutagenesis Kit (TOYOBO, SMK-101), respectively. These constructs were transformed into *alr1-1* by *Agrobacteria* strain GV3101-mediated transformation. For generating *ALR1ox1* and *ox2*, the full-length *ALR1* coding sequence was cloned into *pCAMBIA1301-35S* and then transformed into WT. For generating *ALR1p:GUS*, the same *ALR1* native promoter was cloned into *pCambia1301-GUS* and then transformed into WT. For *RbohD* transgenic complementation test, a full-length *RbohD* coding sequence (2766 bp) driven by its native promoter (2448 bp) was cloned into *pCambia1301* to generate *RbohDp:RbohD*. The *RbohDp:RbohD*^*S39D*^ construct was generated by site-directed mutagenesis in *RbohDp:RbohD*. These constructs were then transformed into *rbohD-1* background. For *RAE1* complementation test, the coding sequences of full-length RAE1 (1998 bp) and mutated RAE1 (RAE1^C364A^) driven by *RAE1* native promoter (1740 bp) were cloned into *pCAMBIA1301* to generate *RAE1p:RAE1* and *RAE1p:RAE1*^*C364A*^, respectively, and were further transformed into *rae1-1*/*GFP-STOP1* to generate *RAE1/rae1-1/GFP-STOP1* and *RAE1*^*C364A*^*/rae1-1/GFP-STOP1* transgenic lines, respectively. For generating *ALR1*^*4C-A*^*/alr1-1/STOP1-GFP* lines, the coding sequence of full-length *STOP1* (1500 bp) fused with *GFP* (720 bp) was cloned into *pCAMBIA1301-35Sp* to generate *35Sp:STOP1-GFP* construct, which was then transformed into Col-0 and *ALR1*^*4C-A*^*/alr1-1*, respectively. To generate *PSK4ox* and *PSK5ox*, the coding sequence of full-length *PSK4* and *PSK5* were cloned into *pCAMBIA1301-35Sp*, respectively, and were further transformed into Col-0. *PSK4ox/alr1-1* and *PSK5ox/alr1-1* were obtained by crossing *PSK4ox* or *PSK5ox* with *alr1-1*, respectively. To generate the *FLAG-ALR1*, *FLAG-RbohD* and *FLAG-RAE1* transgenic lines, the full-length coding sequences of *ALR1*, *RbohD* and *RAE1* were respectively cloned into the *pCAMBIA1300-35S-3×FLAG* plasmid. The resulting constructs were introduced into WT and *alr1-1* plants by agrobacterium-mediated transformation. Primers used are listed in Supplementary Table S2.

### Al treatment

For phenotypical analysis under Al treatment, a soaked gel medium was used to evaluate Al sensitivity as previously described with minor modification.^16^ 1/6 MS agar medium plus 0.5% sucrose and 0.8% agar (Sigma-Aldrich, A7002) was soaked with 25 mL of the same nutrient medium with or without 1 mM AlCl_3_ at pH 3.6. After 2 d of soaking, the solution was removed, and seeds were grown on the agar medium in plates for 7–10 d. For metal ion treatments in hydroponic culture, seeds were sowed on 1/8 MS with or without 2–5 µM AlCl_3_, 2 µM CuSO_4_, 2 µM CdCl_2_, 10 µM LaCl_3_, 10 µM FeCl_3_ at pH 5.2. The seedlings were then photographed and the root lengths were measured with ImageJ software. For data presentation, the average length of each genotype under the control condition was set to 100%, and the relative root length was expressed as a percentage (root length with Al treatment/root length without Al × 100), which was used to evaluate Al sensitivity.

A library of RLK T-DNA insertion mutants (including ~300 RLK mutants) was collected in the preliminary study. The Al sensitivity of each mutant was detected on the Al-soaked gel medium as described above, and *alr1-1* was isolated.

For other analysis under Al treatment, roots were pretreated with 0.5 mM CaCl_2_ (pH 4.5) solution for 2 h, and then treated with the same solution with or without AlCl_3_ for the indicated time.

For soil experiments, the acid soil (pH 4.3) used in this study was a mix of normal soil (pH 6.5) and acid soil (pH 4.0). To minimize the difference in nutrient levels in the two soils, the full-strength nutrient solution (pH 4.3) was used to irrigate the two soils. Furthermore, the *almt1* mutant was used as a positive control showing the Al toxicity in acid soils.

### Determination of root Al content

Root samples from 1-week-old seedlings following 24 h Al (50 µM) treatment were used for Al content measurement. The Al content in the whole root, root cell sap, or root cell wall was determined as previously described.^53^

### Detection of root organic acid anion exudation

Both 4-week-old plants and 1-week-old seedlings were used for determination of root organic acid anion secretion. For 4-week-old plants, the secretion of organic acid anions was detected as previously described.^48^ For 1-week-old seedlings, they were transferred to 6-well plate (100 seedlings per well) and pretreated with 0.5 mM CaCl_2_ solution containing 1% sucrose for 2 h at pH 4.5, and then were treated with 4 mL of the same solution with or without 50 µM AlCl_3_ for 24 h with constant shaking on a rotary shaker (50 rpm) at room temperature in darkness. Malate and citrate concentrations in exudation media were determined by NAD/NADH cycling-coupled enzymatic method as described previously.^54^

### Confocal microscopic analysis

For observation of Al-induced GFP-STOP1 fluorescence, 1-week-old *STOP1p:GFP-STOP1* seedlings in various genetic backgrounds were treated with or without 50 µM AlCl_3_ in 0.5 mM CaCl_2_ solution (pH 4.5) for 1.5 h. For chemical treatment, seedlings were treated with 50 µM AlCl_3_ in 0.5 mM CaCl_2_ solution (pH 4.5) plus 50 µM DPI, 50 µM AlCl_3_ plus 10 mM imidazole, 50 µM AlCl_3_ plus 50 µM NAC, 10 µM MV and 200 µM H_2_O_2_ for 1.5 h, respectively. GFP fluorescence was detected and photographed using a confocal laser scanning microscope (Zeiss) with the same parameter setting. For ROS visualization, 1-week-old seedlings were pre-treated with 100 µM NAC in 0.5 mM CaCl_2_ solution (pH 4.5) for 8 min (to remove background ROS), and were transferred into fresh 0.5 mM CaCl_2_ solution with or without 15 µM AlCl_3_ and 10 µM H_2_DCF-DA for 10 min. The fluorescence was then detected and photographed using a fluorescence microscope (Nikon). The fluorescence intensities of GFP and ROS were analyzed using software ImageJ. The average fluorescence intensity of GFP-STOP1 or ROS in WT under Al treatment was set to 100%, and the relative fluorescence intensity was expressed as a percentage (fluorescence intensity in different genotypes under Al treatment/fluorescence intensity in WT under Al treatment × 100, or fluorescence intensity in WT under other treatments/fluorescence intensity in WT under Al treatment × 100).

### Gene expression analysis

For real-time qPCR, total RNAs were extracted from the roots of 1-week-old seedlings with or without 25 µM Al treatment for 6 h, and converted to cDNAs as previously described. RT-qPCR analysis was carried out using the SYBR Green Realtime PCR Master Mix (TOYOBO) on a Roche LightCycler480 real-time qPCR system following the manufacturer’s instructions. Transcript levels of each mRNA were determined and normalized with the level of *UBQ10* mRNAs using the ΔCt method.^55^

### RNA-sequencing (RNA-seq) assay

For RNA-seq assay, total RNAs were extracted from the roots of 1-week-old WT, *alr1-1* and *stop1* seedlings with or without 200 µM Al treatment for 6 h in 0.5 mM CaCl_2_ solution at pH 5.2. Three biological replicates were done. The RNA-seq analysis was performed by Novogene Science and Technology Co., Ltd. (Tianjin, China) using the Illumina NovaSeq 6000 Sequencing System (150 bp paired-end reads; 6 G). The differentially expressed genes were identified using edge R (| log_2_Fold Change | > = 0.6, FDR < 0.05).

### Yeast two-hybrid assay

A split-ubiquitin membrane yeast two-hybrid system was used as previously described.^56^ *pPR3-N-RbohD* (AD-RbohD) and *pPR3-N-STOP1* (AD-STOP1) constructs were either individually transformed or co-transformed with *pBT3-STE-ALR1* (BD-ALR1) into NMY51 yeast competent cells according to the manufacturer’s instructions (Clontech). Transformed yeast cells were grown in SD (–Leu/Trp) liquid media to an OD_600_ of 0.1 and diluted in a 10× dilution series with 0.9% NaCl, and were further spotted on SD double dropout (–Leu/Trp) and SD quadruple dropout (–Ade/His/Leu/Trp) media plates.

### Split luciferase (LUC) complementation assay

The *35* *S:cLUC-RbohA/C/D/E/F* and *35* *S:ALR1-nLUC* constructs were transferred into the *Agrobacterium* GV3101 lines individually, and the GV3101 lines were co-infiltrated into the *N. benthamiana* leaves. Luciferase imaging was performed 48 h post infiltration using a NightShade LB 985 in vivo Plant Imaging System with a CCD camera.

### Transient expression in protoplasts

Mesophyll cell protoplasts were isolated mainly as previously described.^57^ The protoplasts that were transformed with the same constructs and were further exposed to different treatments were from the same pool; otherwise, they were from independent pools. For independent transformation, we used a *35* *S:LUC* plasmid as an internal control to monitor the transformation efficiency.

To assess RAE1 and RAE1 mutants-mediated STOP1 protein degradation, protoplasts were transfected with *HBT-35S:STOP1-GFP* and *pUC35S-Flag-RAE1*or *pUC35S-Flag-RAE1m* (including *RAE1*^*C160A*^, *RAE1*^*C218A*^, *RAE1*^*C262A*^, *RAE1*^*C288A*^, *RAE1*^*C339A*^, *RAE1*^*C365A*^, *RAE1*^*C391A*^, *RAE1*^*C507A*^, *RAE1*^*C364A*^, *RAE1*^*9C-A*^ and *RAE1*^*8C-A*^). After 12 h incubation at room temperature, the protoplasts were harvested and the STOP1-GFP fluorescence signal was detected using a fluorescence microscope (Nikon). For MV treatment, 10 µM MV was added to the protoplasts after transfection for 6 h.

For immunoblot assay, 1 mL of protoplasts were transfected with 50 µg *HBT-35S:STOP1-GFP* and 50 µg *pUC35S-Flag-RAE1* or *pUC35S-Flag-RAE1*^*C364A*^ or *pUC35S-Flag-empty*. The protoplasts were harvested and lysed in 300 µL of lysis buffer (50 mM Tris-HCl, pH 7.4, 5 mM EDTA, 150 mM NaCl, 1% Trition X-100, 0.1% SDS, 0.5% sodium deoxycholate, 1× protease inhibitor cocktail). After centrifugation at 13,000× *g* for 10 min, 20 µL of supernatants were separated on 10% SDS-polyacrylamide gels, and the proteins were then transferred to PVDF membrane by wet electroblotting and were further detected by α-GFP (ABclonal) and α-actin (ABclonal) antibodies with dilution of 1:5000, respectively.

To assess RAE1 and RAE1^C364A^-mediated ubiquitination of STOP1, 1 mL of protoplasts were transfected with 50 µg *HBT-35S:STOP1-GFP* and 50 µg *pUC35S-Flag-RAE1* or *pUC35S-Flag-RAE1*^*C364A*^ or *pUC35S-Flag-empty*. The protoplasts were harvested and lysed in 300 µL of lysis buffer. After centrifugation at 13,000× *g* for 10 min, 50 µL of the supernatants were kept as input, while the rest was incubated with 20 µL of GFP-Trap (ChromoTek) for 2 h. After the beads were washed three times with TBS buffer, the ubiquitinated conjugates were detected by immunoblot with α-GFP (1:5000; ABclonal) and α-Ub (1:5000; Ubbiotech) antibodies. The input was detected with α-GFP (1:5000; ABclonal) and α-FLAG (1:5000; ABclonal) antibodies.

### BiFC assay

To generate constructs for BiFC assay, the full-length *ALR1*, chimeric ALR1 (comprised of ALR1 extracellular and transmembrane domains (1–680 aa) and the PSY1R cytoplasmic domain (740–1095 aa)), *RbohD*, the full-length BAK1 and the truncated BAK1 (BAK1^ED^, comprised of BAK1 extracellular and transmembrane domains (1–249 aa)) fragments were cloned into *pUC35S-nYFP* or *pUC35S-cYFP* (BIOGLE GeneTech) vectors to obtain ALR1-nYFP, ALR1^ED^-TM-PSYR1^CD^-nYFP, RbohD-cYFP, BAK1-cYFP, and BAK1^ED^-TM-cYFP, respectively. These plasmids were selectively co-transferred into the *Arabidopsis* mesophyll protoplasts. Cells were incubated in W5 solution for 10–12 h, and the expression of YFP was detected using a confocal laser scanning microscope (Zeiss). For AlCl_3_ or LaCl_3_ treatment, cells from the same pool were treated with 50 µM AlCl_3_ or 50 µM LaCl_3_ for 10 min before fluorescence detection. The nYFP or cYFP empty plasmids were used as negative controls.

### NADPH oxidase activity assay

For NADPH oxidase activity assay, 1-week-old *Arabidopsis* WT, *alr1-1*, *rbohD-2*, *RbohD*^*S39A*^*/rbohD-2*, *ALR1*^*CD*^*/alr1-1* and *ALR1*^*4C-A*^*/alr1-1* grown on 1/2 MS media were treated with or without 50 µM AlCl_3_ in 0.5 mM CaCl_2_ solution (pH 4.5) for 1.5 h. The seedlings were harvested and lysed in 1 mL of RIPA buffer (50 mM Tris-HCl pH 7.4, 5 mM EDTA, 150 mM NaCl, 1% Triton X-100, 0.1% SDS, 0.5% sodium deoxycholate, 1× protease inhibitor cocktail) and incubated for 1.5 h at 4 °C. Then the lysed cells were clarified by centrifugation at 13,000× *g* for 10 min, and the supernatants were used to detect NADPH oxidase activity as previously described.

### Recombinant protein expression and purification

For recombinant protein expression in *E.coli*, *ALR1*^*CD*^ (2041–3027 bp), *RbohD*^*N*^ (1–1130 bp), and their mutant variants (*ALR1*^*CD-K762R*^, *ALR1*^*CD-C742A*^, *ALR1*^*CD-C768A*^, *ALR1*^*CD-C852A*^, *ALR1*^*CD-C939/944A*^, *ALR1*^*CD-C985/987A*^, *ALR1*^*CD944A*^, *ALR1*^*CD3C-A*^, *ALR1*^*CD4C-A*^, *TF-RbohD*^*N-S39A*^ and *TF-RbohD*^*N-S152A*^) were amplified and respectively cloned into pCold-TF (TAKAR) vector containing a His-TF-Tag sequence (encoding a 48 kDa chaperone helping decrease protein misfolding). The PCR fragment of *BAK1*^*CD*^ (cytoplasmic domain, 76–1971 bp) was cloned into both pCold-TF and GST-tagged vectors. These plasmids were transformed into *E. coli* BL21 CondonPlus (DE3) strains. Cultures were grown at 37 °C until OD_600_ = 0.4–0.6, and protein expression was induced by 1 mM IPTG at 16 °C for 16 h. After induction, the bacteria were collected by centrifugation at 5000× *g* and stored at –80 °C until use.

The His-tagged recombinant proteins were purified using Ni-NTA superflow column (QIAGEN, 30622) according to the manufacturer’s instructions. The purified proteins were finally eluted by 3 mL elution buffer (50 mM Tris-HCl, 300 mM NaCl, 250 mM imidazole, 2 mM DTT, 1× protease inhibitor cocktail). The protein after dialysis was aliquoted and stored at –80 °C until use.

### Phosphorylation assay

ATP-γ-S-dependent in vitro phosphorylation assay was performed as described previously.^58^ The phosphorylation was detected by α-Thiophosphate ester antibody (1:5000; Abcam, ab92570). For pS39 antibodies-dependent in vitro phosphorylation assay, the recombinant proteins were incubated in kinase reaction buffer (20 mM HEPES pH 7.4, 10 mM MgCl_2_, 1 mM DTT, and 20 mM ATP) with or without AlCl_3_ (0, 1, 10, 50 and 100 nM) for 30 min at 37 °C and stopped by adding 5× SDS loading buffer. The samples were then separated by 10% SDS-PAGE, and the phosphorylation of *His-RbohD*^*N*^ was detected by immunoblot with pS39 antibodies (1:5000). The pS39 antibodies were generated as described previously.^32^

For in vivo phosphorylation assay, 1-week-old *FLAG-RbohD*/WT and *FLAG-RbohD*/*alr1-1* transgenic lines were treated with or without 50 µM AlCl_3_ in 0.5 mM CaCl_2_ solution (pH 4.5) for 2, 5, 10 and 30 min, respectively. The seedlings were harvested and lysed in 1 mL of RIPA buffer. The FLAG-RbohD proteins were immune-precipitated by anti-Flag magnetic beads (Med Chem Express, HY-K0207), and the phosphorylation was detected by immunoblot with pS39 antibodies (1:5000). The total proteins were detected with α-Flag antibody (1:5000; ABclonal).

### BIAM labeling assay

For in vitro BIAM labeling, 30 µL of recombinant proteins were treated with 0, 100 and 500 µM H_2_O_2_ and incubated in labeling buffer (50 mM MES, 100 mM NaCl, 1% Triton X-100, 100 µM BIAM, pH 6.5) at room temperature for 30 min. The proteins were precipitated, re-dissolved, and separated by SDS-PAGE as previously described.^34^ BIAM-labeled proteins were detected by immunoblot with α-biotin HRP-Linked antibody (1:5000; Cell signaling, 7075 S). The total His-STOP1 and His-RAE1 proteins were detected with α-His antibody (1:5000; ABclonal).

For in vivo BIAM labeling, 1-week-old transgenic *Arabidopsis FLAG-RAE1*/WT- and *FLAG-RAE1*/*alr1-1* grown on 1/2 MS media were treated with or without 75 µM AlCl_3_ in 0.5 mM CaCl_2_ solution (pH 4.5) for 0.5 and 1 h, respectively. The seedlings were harvested and lysed in 1 mL of RIPA buffer. The FLAG-RAE1 proteins were immune-precipitated by anti-Flag magnetic beads at 4 °C. The magnetic beads were washed four times, and then incubated with BIAM-labeling buffer at room temperature in the dark for 30 min with constant shaking. BIAM-labeled proteins were finally detected by immunoblot with α-biotin HRP-Linked antibody (1:5000; Cell signaling, 7075 S). The total proteins were detected with α-Flag antibody (1:5000; ABclonal).

### Mass spectrometric analysis for BIAM modification

The purified His-RAE1 protein labeled with BIAM was digested in gel by trypsin, and then analyzed by LC-MS/MS analysis as previously described.^59^ The molecular weight for BIAM modification on Cys is ~326.14 kD.

### Co-IP assay

For Co-IP assays, 3 mL of protoplasts from *FLAG-ALR1*/*WT* were transfected with 200 µg *pUC35S-YFP-BAK1* or *pUC35S-YFP-empty*. After 12 h incubation at room temperature, 50 µM of AlCl_3_ was added to the protoplasts for 10 min. The protoplasts were then harvested and lysed in 500 µL IP buffer (20 mM Tris-HCl, pH 7.4, 1 mM EDTA, 100 mM NaCl, 1% NP-40, 0.25% sodium deoxycholate, 1× protease inhibitor cocktail). After centrifugation at 13,000× *g* for 10 min, 40 µL of supernatants were kept as input controls, whilst the rest was incubated with 25 µL of GFP-Trap (ChromoTek) for 2 h. After the beads were washed five times with TBS buffer, the proteins were eluted by adding 60 µL 2× SDS sampling buffer at 95 °C for 5 min. Then the eluted proteins and input controls were further detected with α-GFP (1:5000; ABclonal) and α-FLAG (1:5000; ABclonal) antibodies.

### MST assay

The MST assay was performed as previously described with minor modification.^60^ The affinity of the purified ALR1^CD^ or its mutant (ALR1^CD4C-A^) with AlCl_3_ or other metal ions (LaCl_3_, CdCl_2_, CeCl_3_, InCl_3_, GaCl_3_, FeCl_3_, MnCl_2_, and CaCl_2_) was measured using the Monolith NT.115 (Nanotemper Technologies). Proteins were first fluorescently labeled using the Monolith Protein Labeling Kit RED-NHS 2nd Generation (Nanotemper Technologies, MO-L011) according to the manufacturer’s protocol, and the labeled protein used for each assay was about 100 nM. A solution of unlabeled metal ions was diluted for appropriate serial concentration gradient. The samples were loaded into MST standard capillaries (Nanotemper Technologies, MO-K022). Measurements were performed in buffer containing 50 mM HEPES, pH 7.4, 150 mM NaCl, 1 mM MgCl_2_, 2 mM DTT, and 0.05% Tween-20, by using medium MST power and 20% LED power. Data were fitted in *Kd* model using MO.Affinity Analysis v2.2.4, and were finally displayed in ΔFnorm normalization.

### CD spectrum

The CD spectrum was obtained using a J-1500 spectropolarimeter (JASCO). The purified protein ALR1^CD^ and ALR1^CD4C-A^ were dissolved in phosphate buffer (pH 7.6) containing 10 mM Na_2_HPO_4_ and 1.8 mM KH_2_PO_4_. Protein concentration was adjusted to 0.2 mg/mL. Measurements were performed at wavelengths ranging from 190 to 260 nm. The cell length was 1 mm, bandwidth was 1 nm, scanning speed was 100 nm/min, response time was 1 s and data pitch was 1 nm.

### Statistical analysis

Independent experiments were performed at least three times, unless indicated otherwise. All data were statistically analyzed using GraphPad Prism 8. The unpaired *t*-test was conducted when two samples were compared. For comparison of multi samples, data were analyzed by two-way ANOVA when two factors were introduced.

### Supplementary information

Fig. S1 Lack of ALR1 reduces Al resistance.

Fig. S2 ALR1 confers specific resistance to Al.

Fig. S3 ALR1 promoted Al resistance is STOP1-dependent.

Fig. S4 RbohD-dependent ROS are required for Al-induced STOP1 accumulation.

Fig. S5 ALR1 phosphorylates RbohD to boost ROS production.

Fig. S6 Phospho-mimicking mutation of Ser39 in RbohD promotes Al resistance and signaling.

Fig. S7 Cys364 is essential for RAE1-mediated STOP1 proteolysis.

Fig. S8 Specific Binding of ALR1CD to Al ions.

Fig. S9 Mutation of Cys939/944/985/987 does not affect the kinase activity or structural integrity of ALR1.

Fig. S10 Analysis of ALR1 mutations in Al resistance and signaling.

Fig. S11 Analysis of ALR1 mutations in Al resistance.

Fig. S12 PSK promotes Al resistance dependently of ALR1.

Fig. S13 Al signaling is separate from PSK signaling.

Table S1 List of ALR1 interactors identified by split-ubiquitin membrane yeast two-hybrid assay

Table S2 Sequence of primers used in this study

### Source data

source data

## Data Availability

The RNA-seq data can be accessed at NCBI with accession number PRJNA913658. All other data needed to evaluate the conclusions in the manuscript are present in the manuscript or the Supplementary information.
