# Supplementary material for: The LRR receptor-like kinase ALR1 is a plant aluminum ion sensor
Source: Cell Res. 2024 Jan 10;34(4):281–94. doi: 10.1038/s41422-023-00915-y (PMC10978910; doi:10.1038/s41422-023-00915-y)
Supplement: Supplementary file 1 — Fig. S1 Lack of ALR1 reduces Al resistance. [file 41422_2023_915_MOESM1_ESM.pdf]

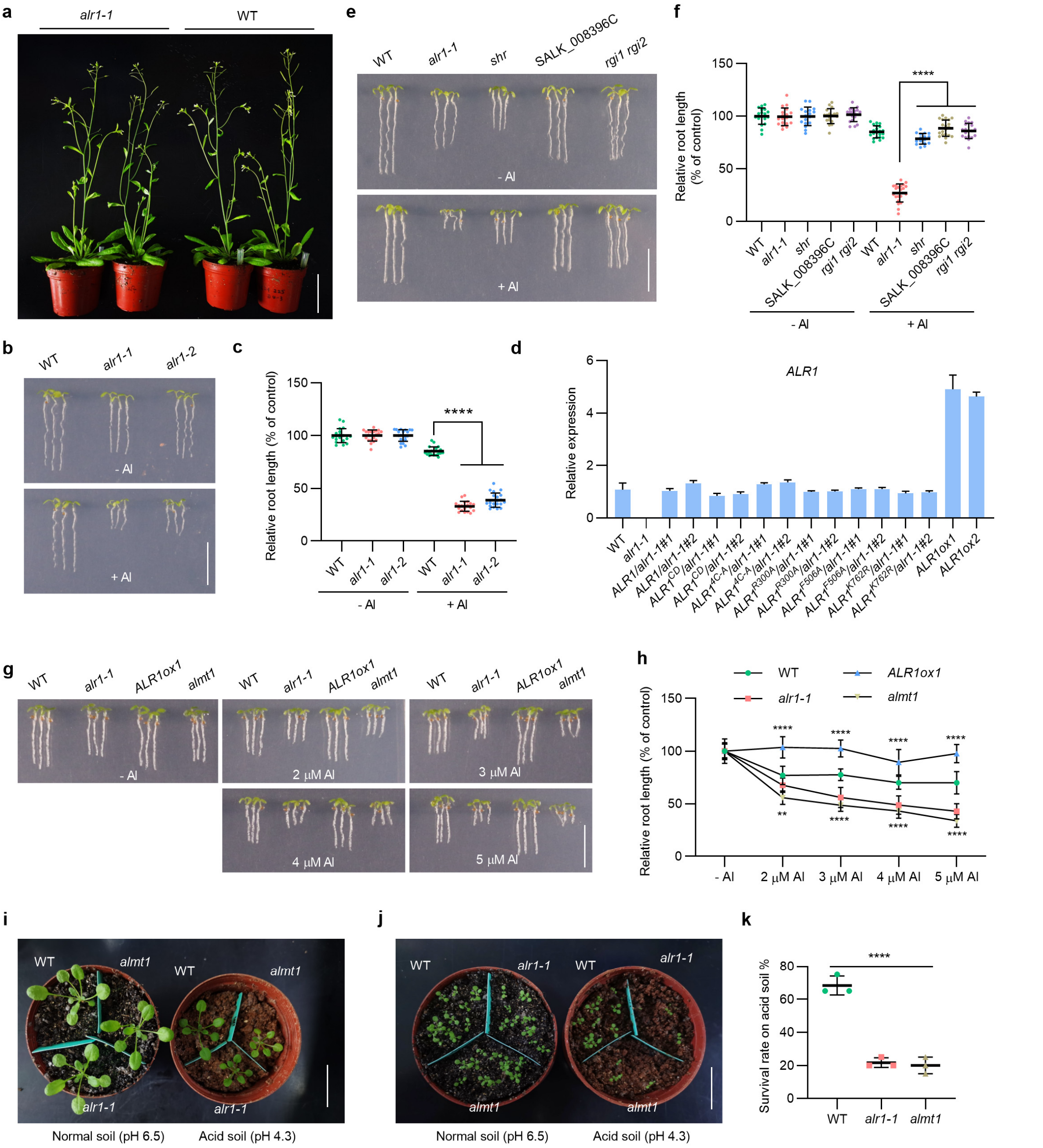

Supplementary information, Fig. S1 Lack of ALR1 reduces Al resistance. a 6-week-old WT and *alr1-1* plants grown on normal soil. **b, c** Root growth under control and Al treatment (**b**), and their relative quantification (**c**) (n = 14-22). The average length of each genotype was set to 100%, and the relative root length was expressed as percentage (root length with Al treatment/root length without Al×100). **d** Expression of *ALR1* in indicated genotypes by RT-qPCR (n = 3). **e, f** Root growth under control and Al treatment (**e**), and their relative quantification (**f**) (n = 16-21). *shr* and *rgi1 rgi2* as described previously[59,60]. **g, h** Root growth under hydroponic culture in the presence or absence of different concentrations of Al (pH 5.2) (**g**), and their relative quantification (**h**) (n = 14-21). **i** 1-week-old seedlings transferred onto the normal (pH 6.5) or acid (pH 4.3) soil for 2

weeks. **j** Seeds sowed on the normal (pH 6.5) or acid (pH 4.3) soil for 10 days. **k** Survival rate on acid soil in (**h**) (n = 3, 20 seedlings per sample). Bars = 5 cm (**a**), 1 cm (**b**, **e**, **g**) and 2 cm (**i**, **j**). All data were analyzed by unpaired t test (**c, f, h, k**) (**P<0.01, ****P<0.0001).
