# Supplementary material for: The LRR receptor-like kinase ALR1 is a plant aluminum ion sensor
Source: Cell Res. 2024 Jan 10;34(4):281–94. doi: 10.1038/s41422-023-00915-y (PMC10978910; doi:10.1038/s41422-023-00915-y)
Supplement: Supplementary file 2 — Fig. S2 ALR1 confers specific resistance to Al. [file 41422_2023_915_MOESM2_ESM.pdf]

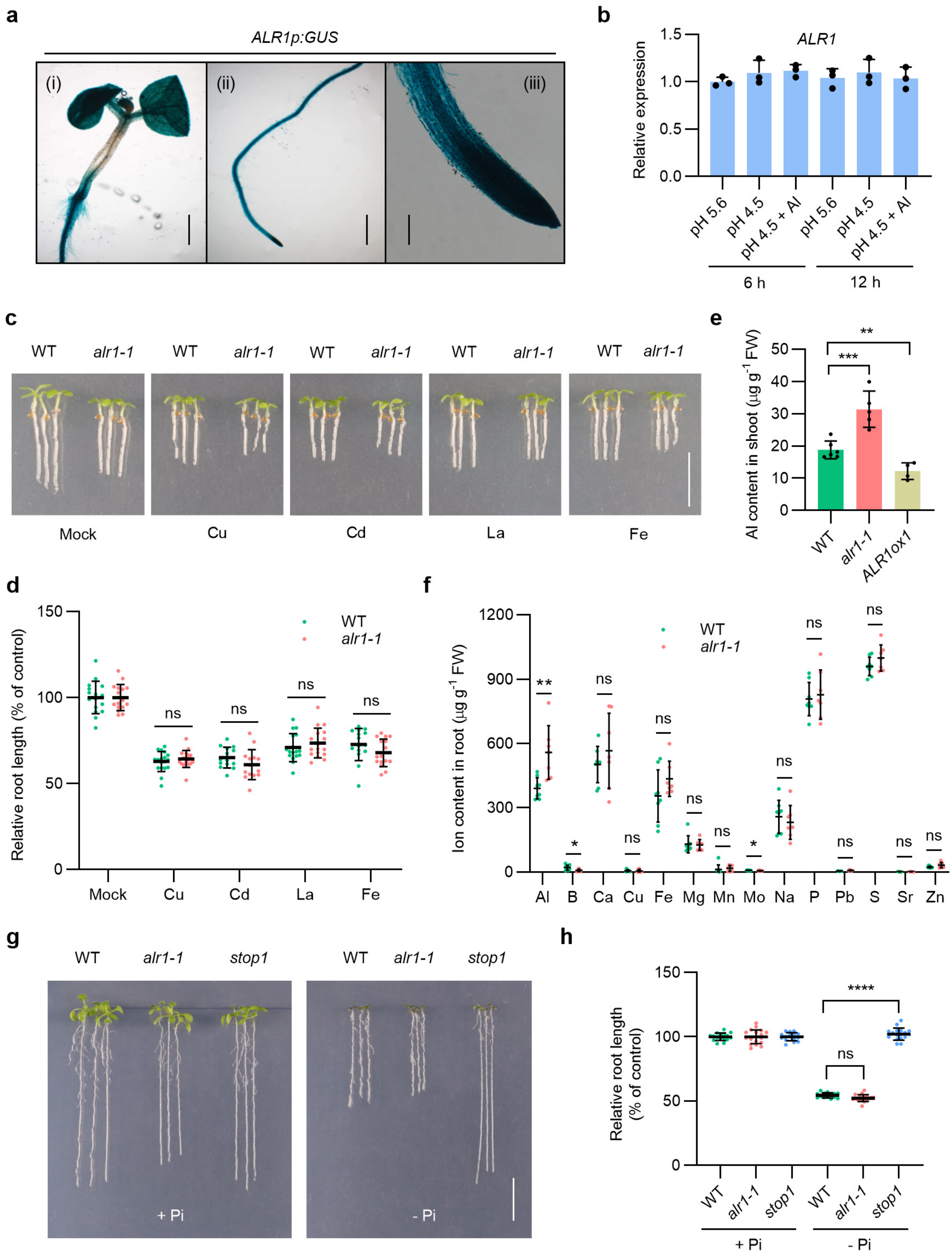

Supplementary information, Fig. S2 ALR1 confers specific resistance to Al. a GUS staining of ALR1p:GUS transgenic line. Bars = 1 mm (in i and ii) and 50 μm in (iii). **b** Expression analysis of *ALR1* in roots under indicated treatments (n = 3). **c, d** Root growth under hydroponic culture in the presence or absence of different metal ions (2 μM Cu, 2 μM Cd, 10 μM La and 10 μM Fe, pH 5.2) (**c**), and their relative quantification (**d**) (n = 14-18). The average length of each genotype was set to 100%, and the relative root length was expressed as percentage (root length with

treatment/root length with control×100). **e** Al content in shoot under Al (25 μM) treatment (n = 4-6). **f** Ion content analysis in whole roots (n = 8 for WT, and 7 for *alr1-1*). **g, h** Root growth on control and phosphate deficient medium (**g**), and their relative quantification (**h**) (n = 14-19). Bar = 1 cm. All data were analyzed by unpaired t test (ns indicates non-significance, *$P<0.05$, **$P<0.01$, ***$P<0.001$, ****$P<0.0001$).
