# Supplementary material for: The LRR receptor-like kinase ALR1 is a plant aluminum ion sensor
Source: Cell Res. 2024 Jan 10;34(4):281–94. doi: 10.1038/s41422-023-00915-y (PMC10978910; doi:10.1038/s41422-023-00915-y)
Supplement: Supplementary file 3 — Fig. S3 ALR1 promoted Al resistance is STOP1-dependent. [file 41422_2023_915_MOESM3_ESM.pdf]

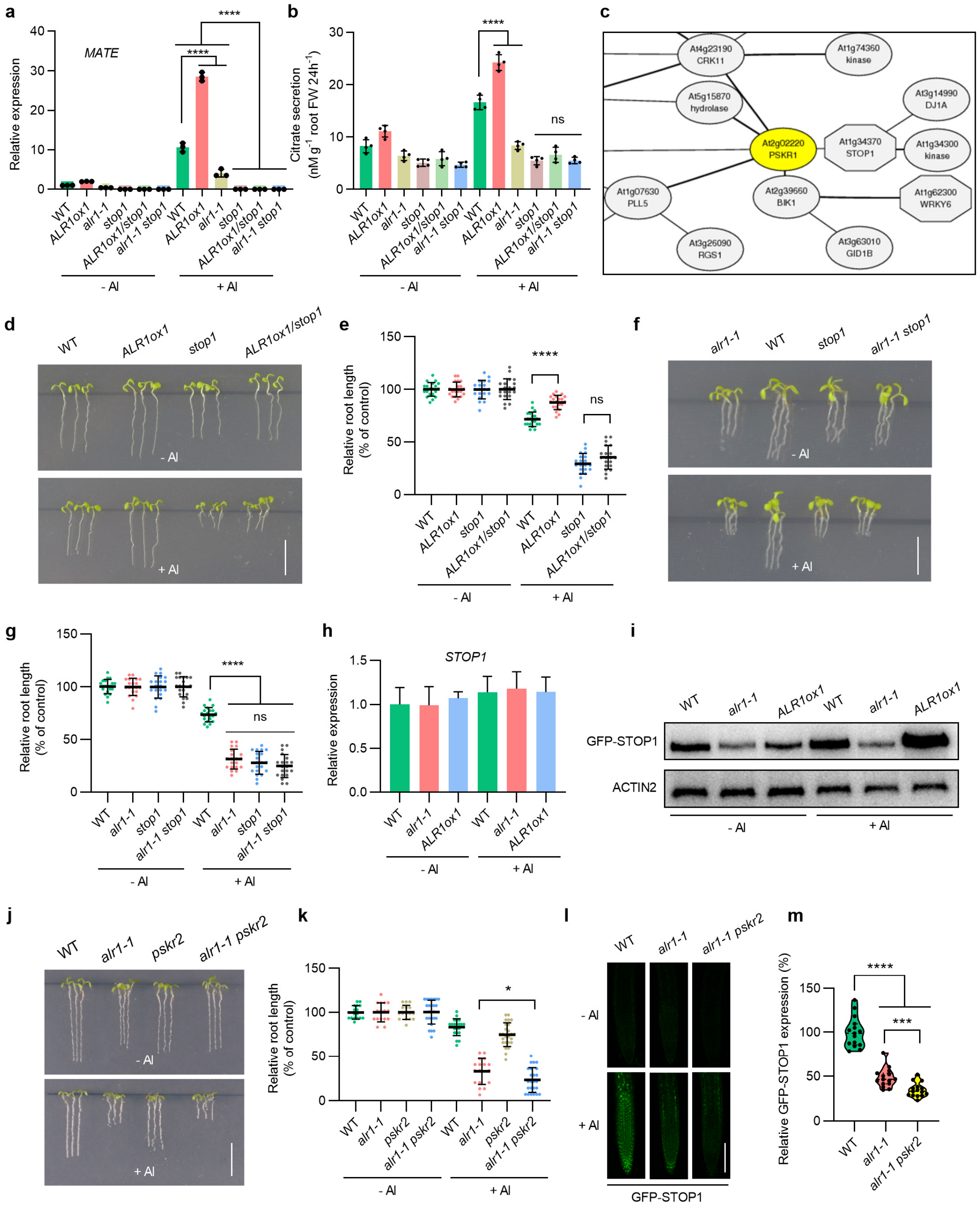

Supplementary information, Fig. S3 ALR1 promoted Al resistance is STOP1-dependent. a Expression of *MATE* in roots under control and Al (25 µM) treatments (n = 3). **b** Citrate secretion from roots under control and Al (50 µM) treatments (n = 4). **c** Co-expression analysis of *ALR1* and *STOP1* in ATTED-II (https://atted.jp/). **d-g** Root growth under control and Al treatments (**d**, **f**), and their relative quantification (**e**, **g**) (n = 20). The average length of each genotype was set to

100%, and the relative root length was expressed as percentage (root length with Al treatment/root length without Al×100). Bars = 1 cm. **h** Expression of *STOP1* in roots under control and Al (25 μM) treatments (n = 3). **i** Detection of GFP-STOP1 in roots using an α-GFP antibody. **j, k** Root growth under control and Al treatment (**j**), and their relative quantification (**k**) (n = 15-25). Bar = 1 cm. **l, m** GFP-STOP1 fluorescence signals in roots (**l**) and their relative quantification under Al treatment (**m**) (n = 20). Bar = 100 μm. All data were analyzed by unpaired t test (**a, b, e, g, k, m**), or two-way ANOVA (**a**) (ns indicates non-significance, **P*<0.05, ****P*<0.001, *****P*<0.0001).
