# Supplementary material for: The LRR receptor-like kinase ALR1 is a plant aluminum ion sensor
Source: Cell Res. 2024 Jan 10;34(4):281–94. doi: 10.1038/s41422-023-00915-y (PMC10978910; doi:10.1038/s41422-023-00915-y)
Supplement: Supplementary file 4 — Fig. S4 RbohD-dependent ROS are required for Al-induced STOP1 accumulation. [file 41422_2023_915_MOESM4_ESM.pdf]

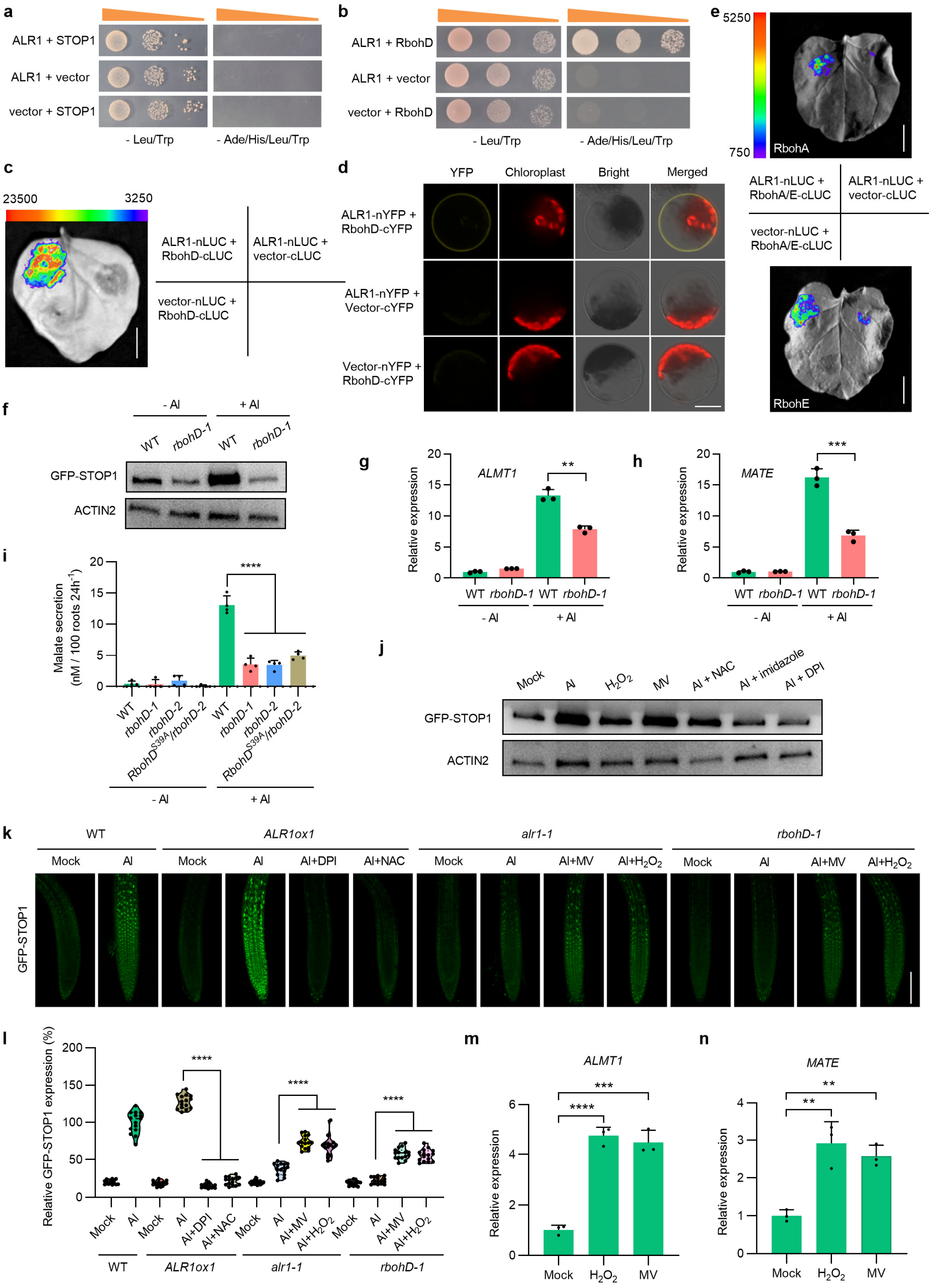
a
ALR1 + STOP1
ALR1 + vector
vector + STOP1
- Leu/Trp
- Ade/His/Leu/Trp
b
ALR1 + RbohD
ALR1 + vector
vector + RbohD
- Leu/Trp
- Ade/His/Leu/Trp
e
5250
750
RbohA
ALR1-nLUC + RbohA/E-cLUC
ALR1-nLUC + vector-cLUC
vector-nLUC + RbohA/E-cLUC
RbohE
c
23500
3250
ALR1-nLUC + RbohD-cLUC
ALR1-nLUC + vector-cLUC
vector-nLUC + RbohD-cLUC
d
YFP
Chloroplast
Bright
Merged
ALR1-nYFP + RbohD-cYFP
ALR1-nYFP + Vector-cYFP
Vector-nYFP + RbohD-cYFP
f
- Al
+ Al
WT
rbohD-1
WT
rbohD-1
GFP-STOP1
ACTIN2
g
ALMT1
Relative expression
WT rbohD-1 WT rbohD-1
- Al
+ Al
h
MATE
Relative expression
WT rbohD-1 WT rbohD-1
- Al
+ Al
i
Malate secretion (nM / 100 roots 24h⁻¹)
WT
rbohD-1
rbohD-2
RbohD^S39A/rbohD-2
- Al
+ Al
j
Mock
Al
H2O2
MV
Al + NAC
Al + imidazole
Al + DPI
GFP-STOP1
ACTIN2
k
WT
ALR1ox1
alr1-1
rbohD-1
Mock
Al
Al+DPI
Al+NAC
Al+MV
Al+H2O2
GFP-STOP1
l
Relative GFP-STOP1 expression (%)
Mock
Al
Al+DPI
Al+NAC
Al+MV
Al+H2O2
WT
ALR1ox1
alr1-1
rbohD-1
m
ALMT1
Relative expression
Mock
H2O2
MV
n
MATE
Relative expression
Mock
H2O2
MV

Supplementary information, Fig. S4 RbohD-dependent ROS are required for Al-induced STOP1 accumulation. a, b Yeast two-hybrid assay showing non-interaction between ALR1 and STOP1 (**a**) and interaction between ALR1 and RbohD (**b**). **c** Split luciferase complementation assay showing the interaction of ALR1 with RbohD. **d** Bimolecular fluorescence complementation (BiFC) assay showing the interaction of ALR1 with RbohD. **e** Split luciferase complementation assay showing ALR1 interaction with RbohA and RbohE. **f** Detection of GFP-STOP1 in roots using an α-GFP antibody. **g, h** Expression of *ALMT1* (**g**) and *MATE* (**h**) in roots under control and Al (25 μM) treatments (n = 3). **i** Malate secretion from roots under control and Al (50 μM) treatments (n = 4). **j** GFP-STOP1 protein accumulation in roots under indicated treatment as was done in Fig. 2e. WB was performed using α-GFP antibody. **k, l** GFP-STOP1 fluorescence signals in roots under indicated treatments (**k**) and their relative quantification (**l**) (n = 20). *GFP-STOP1/*WT seedlings were under control and 50 μM Al treatment. *GFP-STOP1/ALR1ox1* was applied with control, 50 μM Al, 50 μM Al plus 50 μM DPI, and 50 μM Al plus 50 μM NAC. *GFP-STOP1/alr1-1* and *GFP-STOP1/rbohD-1* seedlings were applied with control, 50 μM Al, 50 μM Al plus 10 μM MV and 50 μM Al plus 200 μM H_2O_2. **m, n** Expression of *ALMT1* (**m**) and *MATE* (**n**) in roots under indicated treatments. Bars = 1 cm (**c**, **e**), 20 μm (**d**), 100 μm (**l**). All data were analyzed by unpaired t test (**g**, **h**, **i**, **k**, **m**, **n**) (***P*<0.01, ****P*<0.001, *****P*<0.0001).
