# Supplementary material for: The LRR receptor-like kinase ALR1 is a plant aluminum ion sensor
Source: Cell Res. 2024 Jan 10;34(4):281–94. doi: 10.1038/s41422-023-00915-y (PMC10978910; doi:10.1038/s41422-023-00915-y)
Supplement: Supplementary file 5 — Fig. S5 ALR1 phosphorylates RbohD to boost ROS production. [file 41422_2023_915_MOESM5_ESM.pdf]

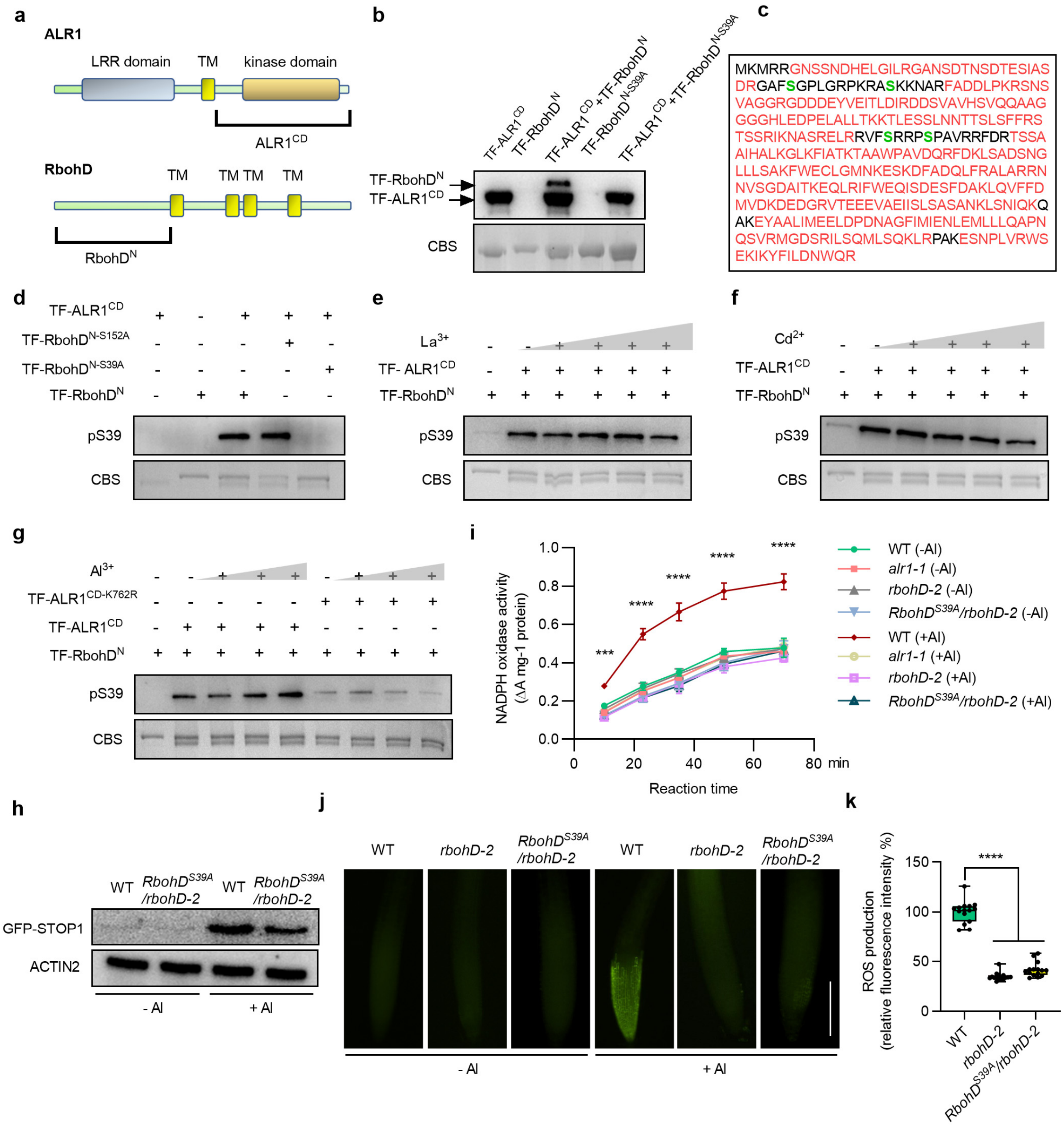

Supplementary information, Fig. S5 ALR1 phosphorylates RbohD to boost ROS production. **a** Diagram of ALR1 cytoplasmic domain and RbohD N-terminal that were used for recombinant expression. **b** Phosphorylation of the recombinant N-terminal of RbohD ($RbohD^{N}$) by the ALR1 cytoplasmic domain ($ALR1^{CD}$) in an ATP-γ-S-dependent *in vitro* kinase assay. CBS indicates coomassie blue staining. **c** Amino acid sequence of the N terminal of RbohD. Peptides detected by MS were highlighted in red. Four undetected Ser residues from MS were indicated in green, including Ser39. **d** Phosphorylation of $RbohD^{N}$ and its mutant forms by $ALR1^{CD}$ detected by pS39 antibodies *in vitro*. **e, f** Phosphorylation of $RbohD^{N}$ by $ALR1^{CD}$ in response to different concentration of $LaCl_3$ (**e**) and $CdCl_2$ (**f**) (0, 1, 10, 50 and 100 nM) *in vitro*. **g** Phosphorylation of $RbohD^{N}$ by $ALR1^{CD}$ or $ALR1^{CD\text{-}K762R}$ *in vitro* (with $AlCl_3$ concentration 1, 10 and 100 nM). The pH of the assays in **d-g** was 7.4. **h** Detection of GFP-STOP1 in roots using an α-GFP antibody. **i**

NADPH oxidase activity of seedlings following 20 min control and Al (50 μM) treatments (n = 3). **j, k** ROS visual signals in roots detected by H_2DCF-DA (**j**), and their relative quantification under 10 min Al treatment (**k**) (n = 20). Bar = 100 μm. Data were analyzed by unpaired t test (**i, j, k**) (*****P*<0.0001).
