# Supplementary material for: The LRR receptor-like kinase ALR1 is a plant aluminum ion sensor
Source: Cell Res. 2024 Jan 10;34(4):281–94. doi: 10.1038/s41422-023-00915-y (PMC10978910; doi:10.1038/s41422-023-00915-y)
Supplement: Supplementary file 6 — Fig. S6 Phospho-mimicking mutation of Ser39 in RbohD promotes Al resistance and signaling. [file 41422_2023_915_MOESM6_ESM.pdf]

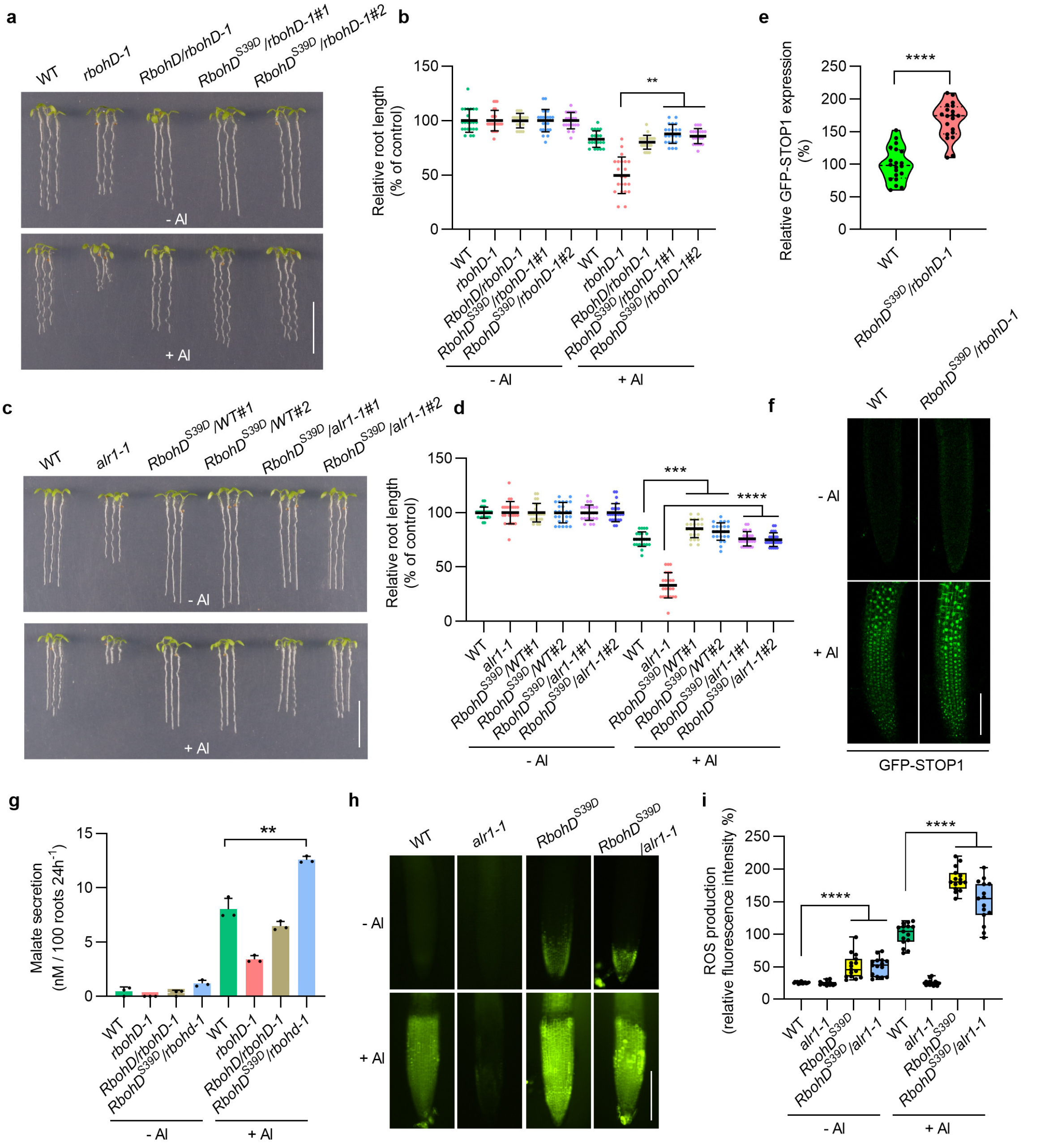

Supplementary information, Fig. S6 Phospho-mimicking mutation of Ser39 in RbohD promotes Al resistance and signaling. a-d Root growth of indicated genotypes under control and Al (1mM) treatments (**a**, **c**), and their relative quantification (**b**, **d**) (n = 20-25). The average length of each genotype was set to 100%, and the relative root length was expressed as percentage (root length with Al treatment/root length without Al×100). **e, f** GFP-STOP1 fluorescence signals in roots (**e**) and their relative quantification (**f**) (n = 20). **g** Malate secretion from roots under control and Al (50 µM) treatment for 24 h (n = 3). **h, i** ROS visual signals in roots under control and Al (15 µM) treatment for 10 min (**h**), and their relative quantification (**i**) (n = 20). Bars = 1 cm (**a**, **c**),

100 μm (**f**, **h**). Data were analyzed by unpaired t test (**b, d, e**, **g, i**) (**$P<0.01$, ***$P<0.001$, ****$P<0.0001$).
