# Supplementary material for: The LRR receptor-like kinase ALR1 is a plant aluminum ion sensor
Source: Cell Res. 2024 Jan 10;34(4):281–94. doi: 10.1038/s41422-023-00915-y (PMC10978910; doi:10.1038/s41422-023-00915-y)
Supplement: Supplementary file 7 — Fig. S7 Cys364 is essential for RAE1-mediated STOP1 proteolysis. [file 41422_2023_915_MOESM7_ESM.pdf]

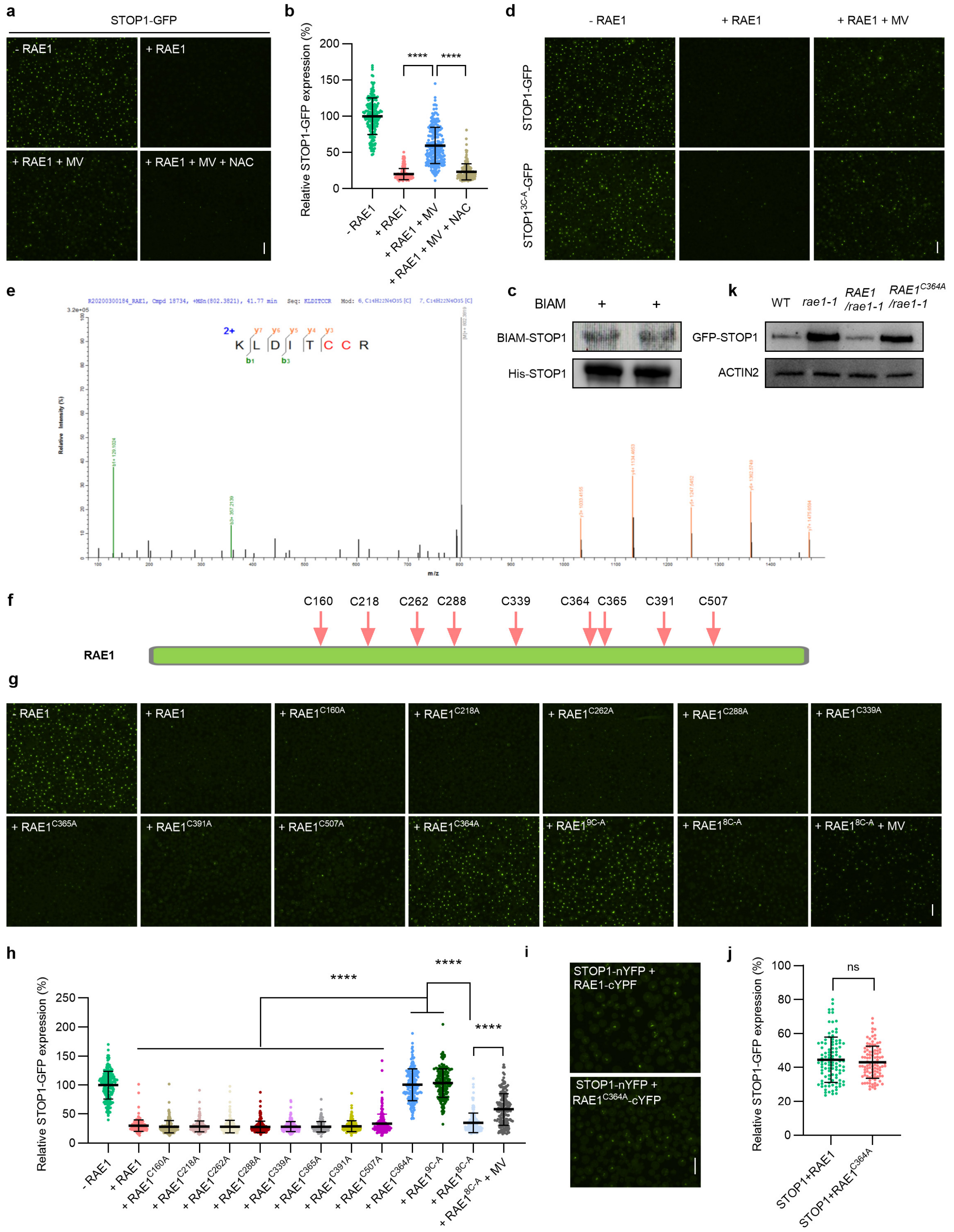

Supplementary information, Fig. S7 Cys364 is essential for RAE1-mediated STOP1 proteolysis. a STOP1-GFP expression in mesophyll protoplasts with or without co-expressing

RAE1 under indicated treatments. **b** Quantification of STOP1-GFP fluorescence intensity in (**a**) (n = 200). **c** *in vitro* labeling of biotin-conjugated iodoacetamide (BIAM) for recombinant His-STOP1. **d** Fluorescence signals of STOP1-GFP and mutant STOP1-GFP (STOP1$^{3C\text{-}A}$-GFP) with or without RAE1 co-expression. **e** Mass spectrometry analysis of the tryptic fragments of His-RAE1 protein labeled with BIAM. **f** Diagram of RAE1 protein. Cys residues modified by BIAM were indicated with arrows. **g, h** STOP1-GFP fluorescence signals in mesophyll protoplasts with or without co-expressing RAE1 or RAE1 mutants (**g**), and their relative quantification (**h**) (n = 200). **i** BiFC mediated detection of interaction between STOP1 and RAE1 or RAE1^{C364A}. **j** Quantification of fluorescence intensity in (**i**) (n = 200). **k** Detection of GFP-STOP1 in roots using an α-GFP antibody. Bars = 500 μm (**a**, **d**, **g**), 50 μm (**i**). Data were analyzed by unpaired t test (**b, f**, **h**) (ns indicates non-significance, ****P<0.0001).
