# Supplementary material for: The LRR receptor-like kinase ALR1 is a plant aluminum ion sensor
Source: Cell Res. 2024 Jan 10;34(4):281–94. doi: 10.1038/s41422-023-00915-y (PMC10978910; doi:10.1038/s41422-023-00915-y)
Supplement: Supplementary file 8 — Fig. S8 Specific Binding of ALR1CD to Al ions. [file 41422_2023_915_MOESM8_ESM.pdf]

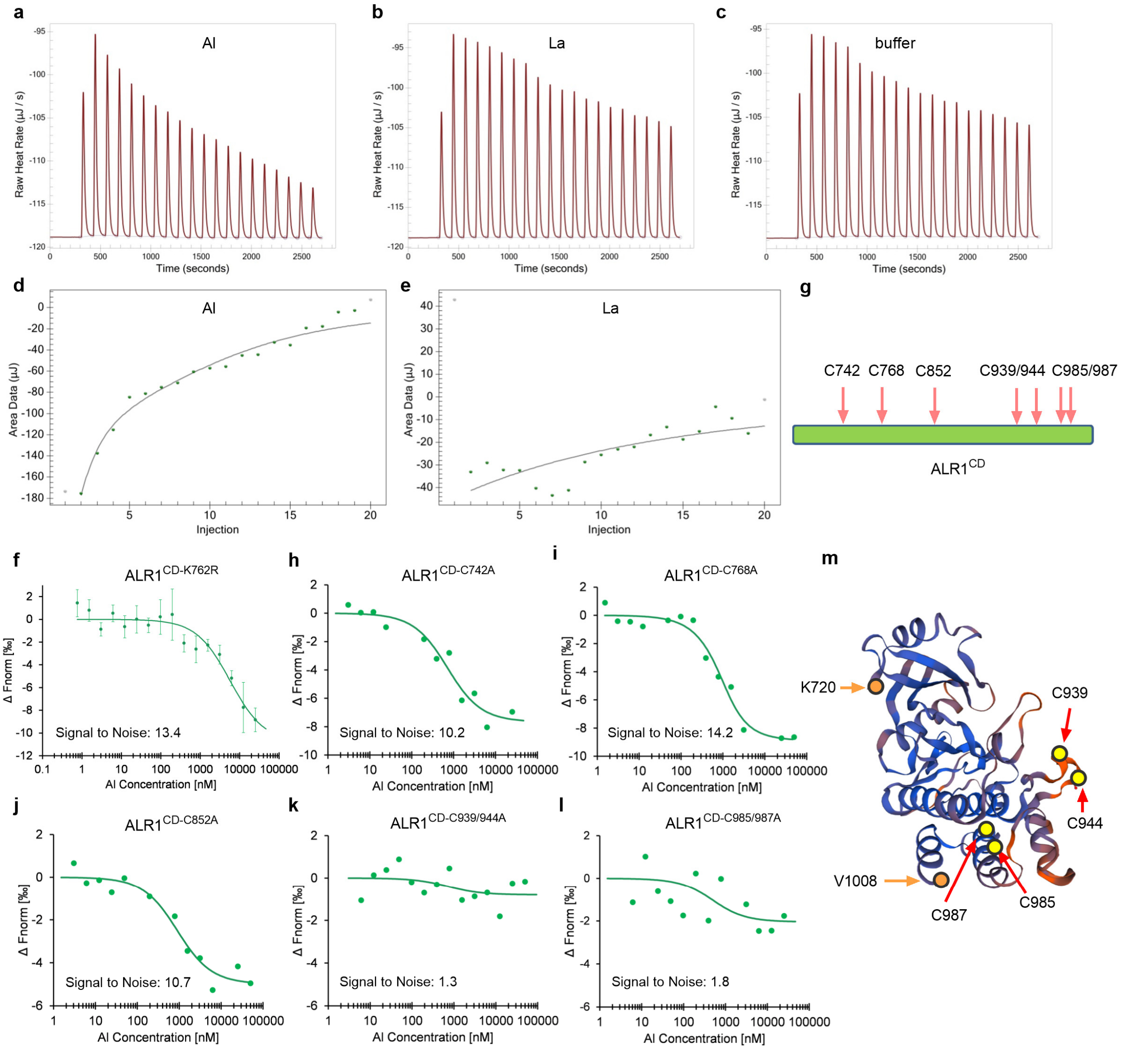

Supplementary information, Fig. S8 Specific Binding of ALR1^{CD} to Al ions. a-c Isothermal titration calorimetry (ITC) data of ALR1KD incubation with Al (**a**), La (**b**) and buffer (**c**, negative control). **d** ITC data (in **a**) were corrected by the negative control (in **c**) and were then best fitted in the model of Multiple Sites with the lowest Kd value of 1 μM. **e** ITC data (in **b**) were corrected by the negative control (in **c**) and were then fitted in the model of Multiple Sites. **f** Quantification of binding affinity between ALR1$^{CD\text{-}K762R}$ and Al ions by MicroScale Thermophoresis (MST) assay (n = 3). **g** Diagram of ALR1 cytoplasmic domain. Cys residues for mutation analysis were indicated with arrows. **h-l** Quantification of binding affinity between mutated ALR1^{CD} and Al ions by MST assay. Each assay was repeated twice with similar result. **m** Modeling of structure of ALR1 cytoplasmic domain. ALR1 cytoplasmic domain was modeled using the SWISS-MODEL server (https://swissmodel.expasy.org/interactive). Four Cys residues required for Al perception were indicated in yellow. The first and last residues of ALR1 cytoplasmic domain were indicated in orange.
