# Supplementary material for: The LRR receptor-like kinase ALR1 is a plant aluminum ion sensor
Source: Cell Res. 2024 Jan 10;34(4):281–94. doi: 10.1038/s41422-023-00915-y (PMC10978910; doi:10.1038/s41422-023-00915-y)
Supplement: Supplementary file 9 — Fig. S9 Mutation of Cys939/944/985/987 does not affect the kinase activity or structural integrity of ALR1. [file 41422_2023_915_MOESM9_ESM.pdf]

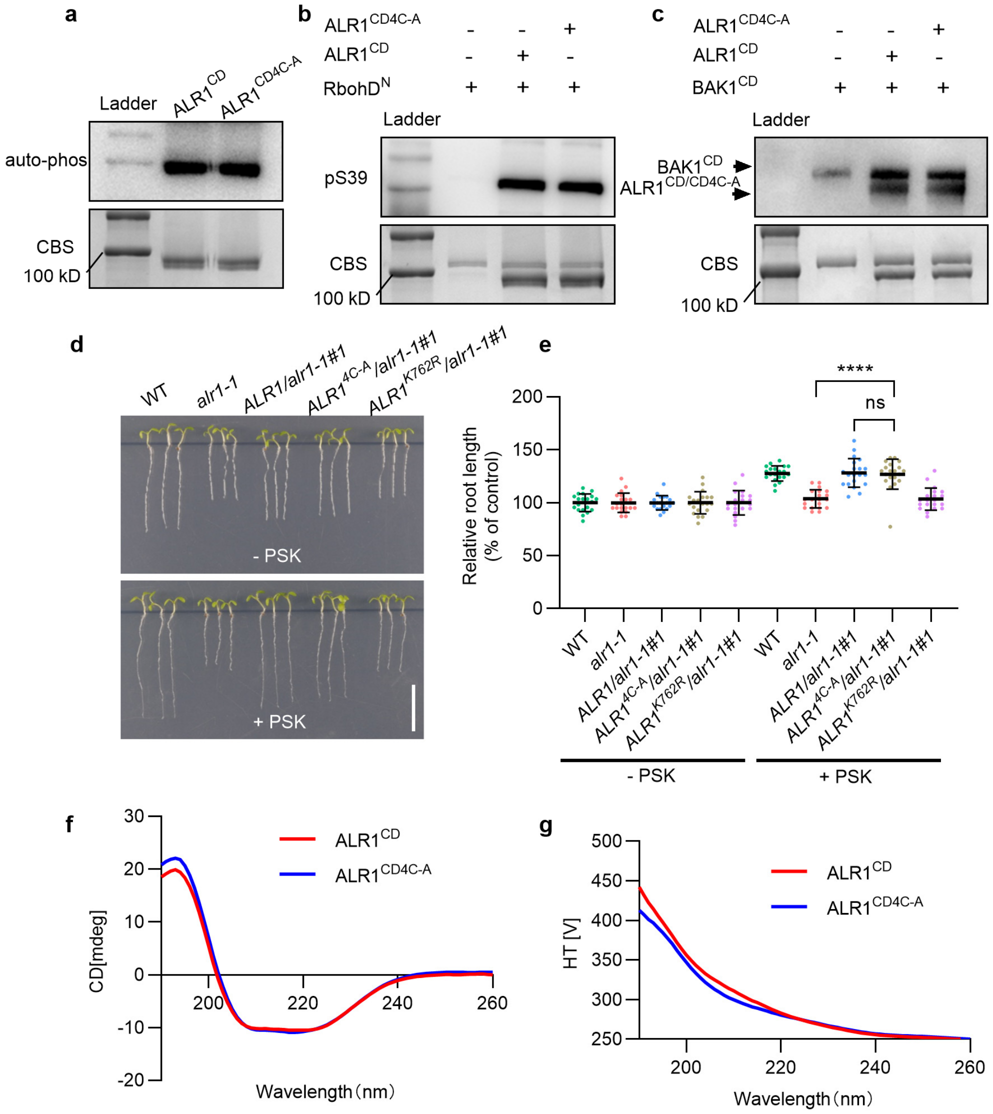

Supplementary information, Fig. S9 Mutation of Cys939/944/985/987 does not affect the kinase activity or structural integrity of ALR1. **a** Auto-phosphorylation of the recombinant ALR1^{CD} and ALR1^{CD4C-A}. **b** Phosphorylation of RbohD by ALR1^{CD} and ALR1^{CD4C-A} was detected by pS39 antibodies. **c** Phosphorylation of BAK1^{CD} by ALR1^{CD} and ALR1^{CD4C-A}. **d** Root growth of indicated genotypes in the absence or presence of 100 nM PSK peptide. Bar = 1 cm. **e** Quantification of relative root growth (in **d**) (n = 20). Data were analyzed by unpaired t test (*****P*<0.0001, ns indicates non-significance). **f**, **g** circular dichroism analysis showing similar structure of ALR1^{CD} and ALR1^{CD4C-A}.
