# Supplementary material for: The LRR receptor-like kinase ALR1 is a plant aluminum ion sensor
Source: Cell Res. 2024 Jan 10;34(4):281–94. doi: 10.1038/s41422-023-00915-y (PMC10978910; doi:10.1038/s41422-023-00915-y)
Supplement: Supplementary file 10 — Fig. S10 Analysis of ALR1 mutations in Al resistance and signaling. [file 41422_2023_915_MOESM10_ESM.pdf]

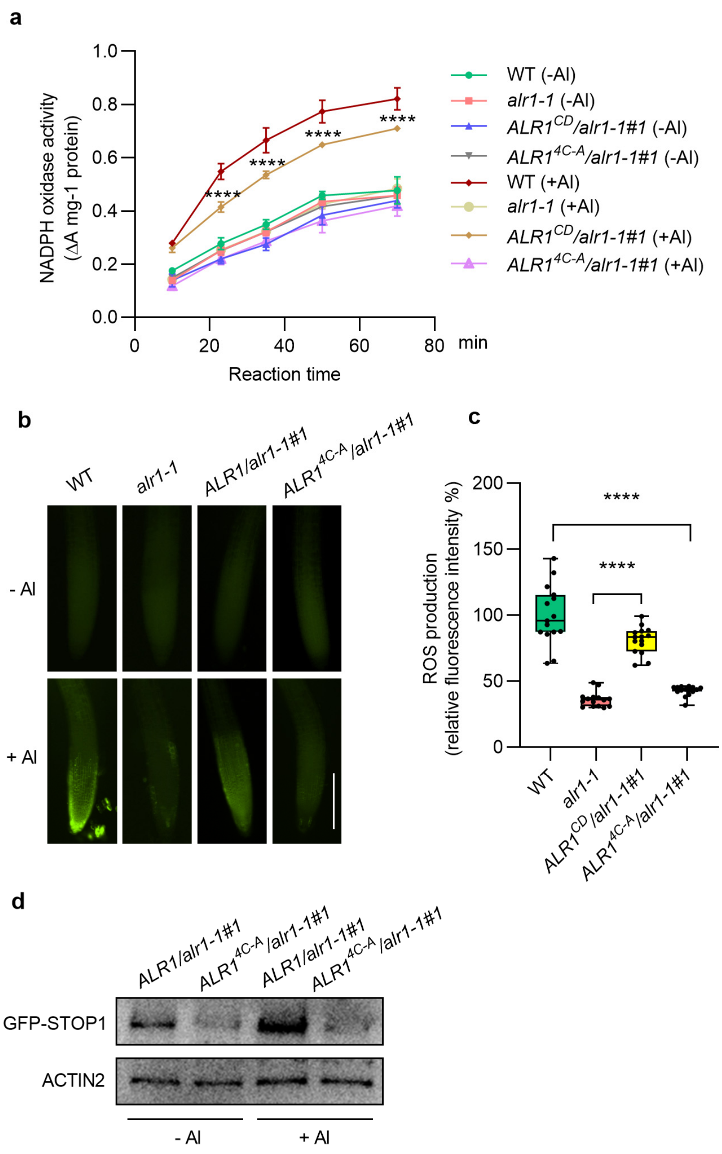

Supplementary information, Fig. S10 Analysis of ALR1 mutations in Al resistance and signaling. a NADPH oxidase activity of seedlings under control and Al treatment for 20 min. The data for WT and *alr1-1* were used same in Fig. S4i. **b, c** ROS visual signals in roots under control and Al (15 µM) treatment for 10 min (**b**), and their relative quantification (**c**) (n = 20). Bars = 100 µm. **d** GFP-STOP1 proteins in root apices of indicated genotypes with or without Al treatment were detected by α-GFP antibody. All data were analyzed by unpaired t test (**a**, **c**) (*****P*<0.0001).
