# Supplementary material for: The LRR receptor-like kinase ALR1 is a plant aluminum ion sensor
Source: Cell Res. 2024 Jan 10;34(4):281–94. doi: 10.1038/s41422-023-00915-y (PMC10978910; doi:10.1038/s41422-023-00915-y)
Supplement: Supplementary file 11 — Fig. S11 Analysis of ALR1 mutations in Al resistance. [file 41422_2023_915_MOESM11_ESM.pdf]

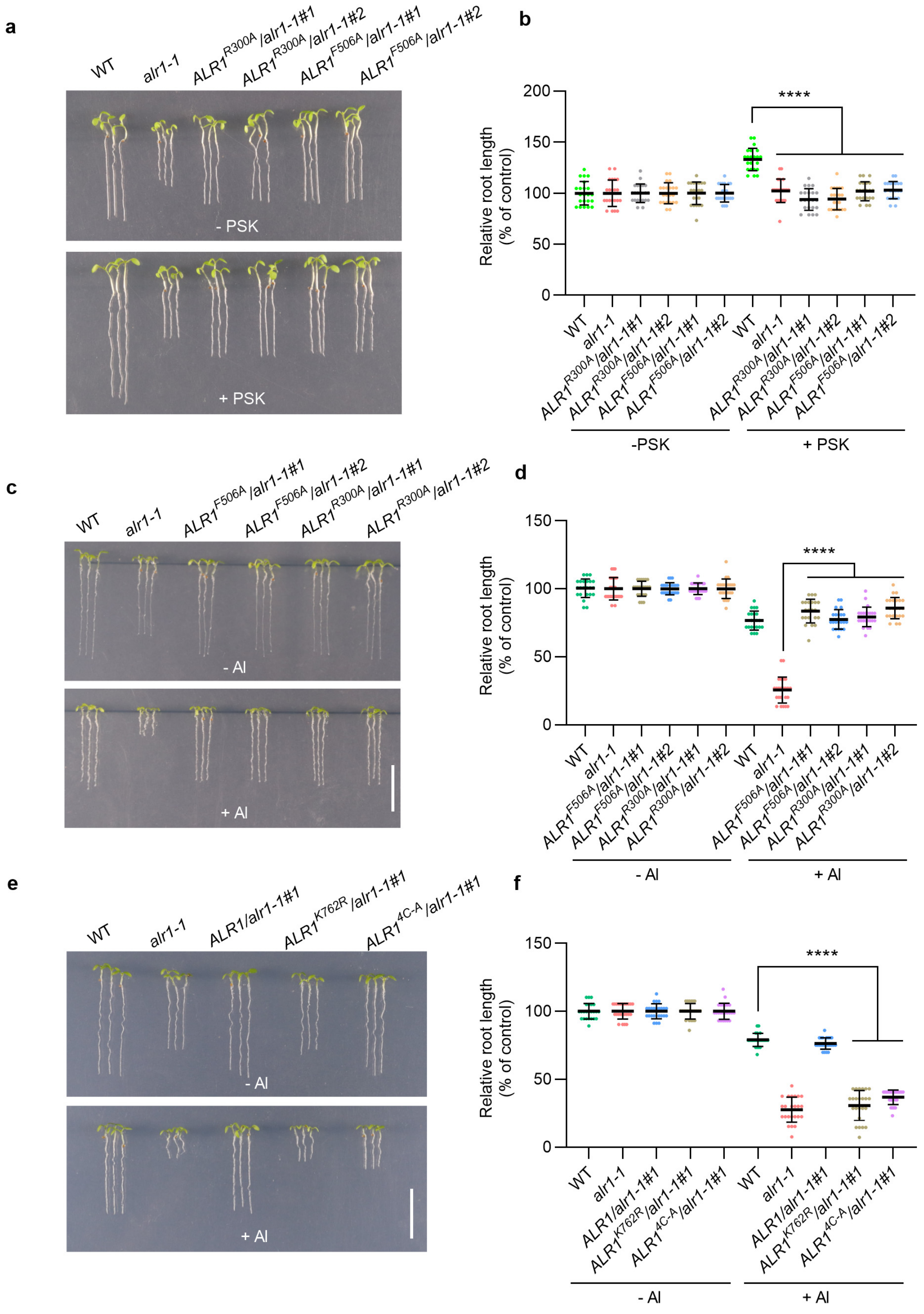

Supplementary information, Fig. S11 Analysis of ALR1 mutations in Al resistance. a Root growth of indicated genotypes under control and PSK (0.1 μM) treatment for 7 days (bar = 1 cm). **b** Quantification of relative root growth in (**e**) (n = 22). The average length of each genotype was

set to 100%, and the relative root length was expressed as percentage (root length with treatment/root length with control×100). **c, e** Root growth of indicated genotypes under control and Al (1mM) treatment for 10 days (bar = 1 cm). **d, f** Quantification of relative root growth in (**c**, **e**) (n = 22-25 for **a**, 21 for **c**). All data were analyzed by unpaired t test (*****P*<0.0001).
