# Supplementary material for: The LRR receptor-like kinase ALR1 is a plant aluminum ion sensor
Source: Cell Res. 2024 Jan 10;34(4):281–94. doi: 10.1038/s41422-023-00915-y (PMC10978910; doi:10.1038/s41422-023-00915-y)
Supplement: Supplementary file 12 — Fig. S12 PSK promotes Al resistance dependently of ALR1. [file 41422_2023_915_MOESM12_ESM.pdf]

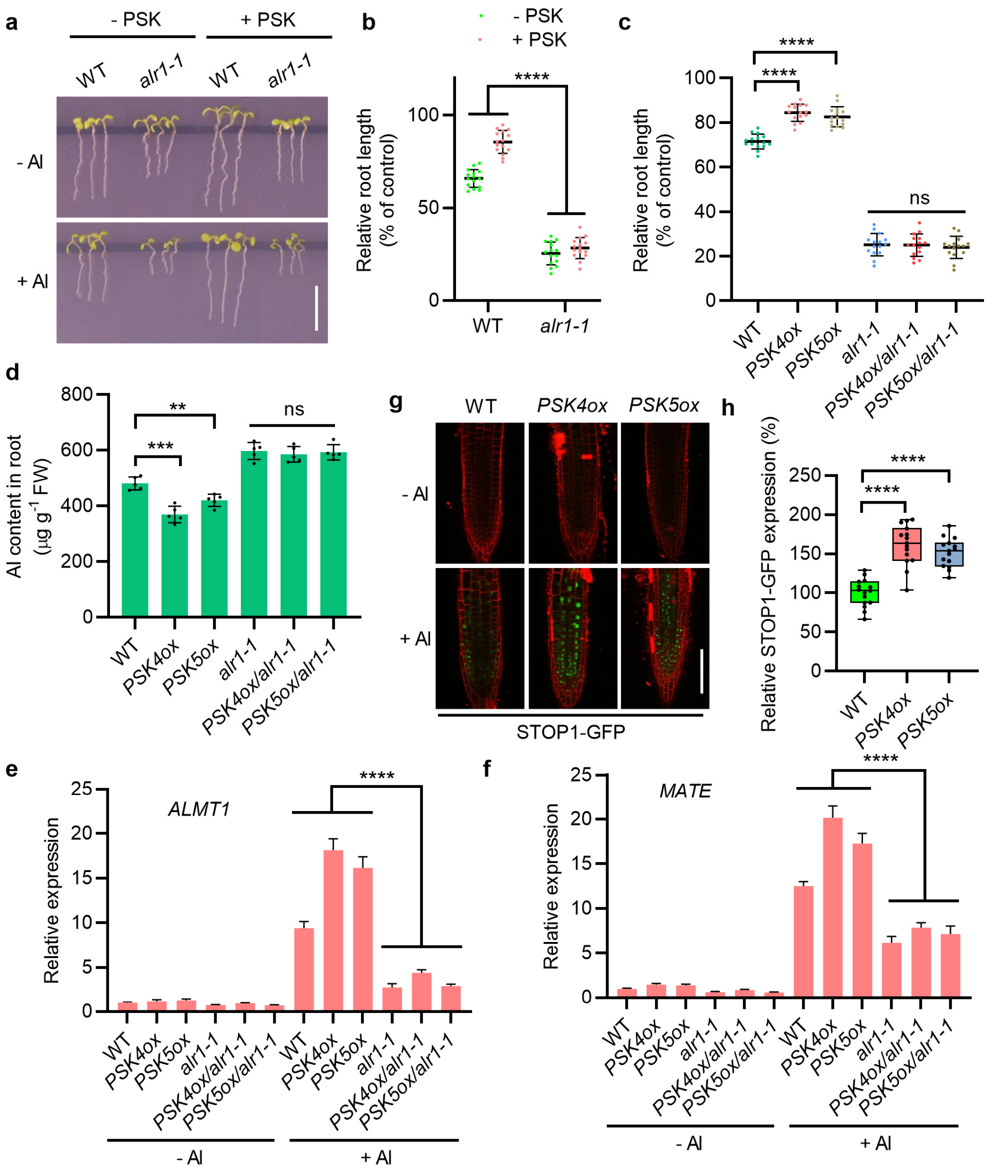

Supplementary information, Fig. S12 PSK promotes Al resistance dependently of ALR1. a, b Root growth of WT and *alr1-1* under control and Al treatments with or without PSK peptide (100 nM) application. The average length of each genotype was set to 100%, and the relative root length was expressed as percentage (root length with Al treatment/root length without Al×100). **c** Root growth of indicated genotypes in response to Al treatment. **d** Al content in roots of indicated genotypes. **e, f** Expression analysis of *ALMT1* and *MATE* in the indicated genotypes under control and Al treatments. **g, h** STOP1-GFP accumulation in the indicated genotypes under Al treatment. Data were analyzed by unpaired t test (**c, d, h**) or two-way ANOVA (in **b**, **e** and **f**) (**P<0.01, ***P<0.001, ****P<0.0001, ns indicates non-significance).
