# Supplementary material for: The LRR receptor-like kinase ALR1 is a plant aluminum ion sensor
Source: Cell Res. 2024 Jan 10;34(4):281–94. doi: 10.1038/s41422-023-00915-y (PMC10978910; doi:10.1038/s41422-023-00915-y)
Supplement: Supplementary file 13 — Fig. S13 Al signaling is separate from PSK signaling. [file 41422_2023_915_MOESM13_ESM.pdf]

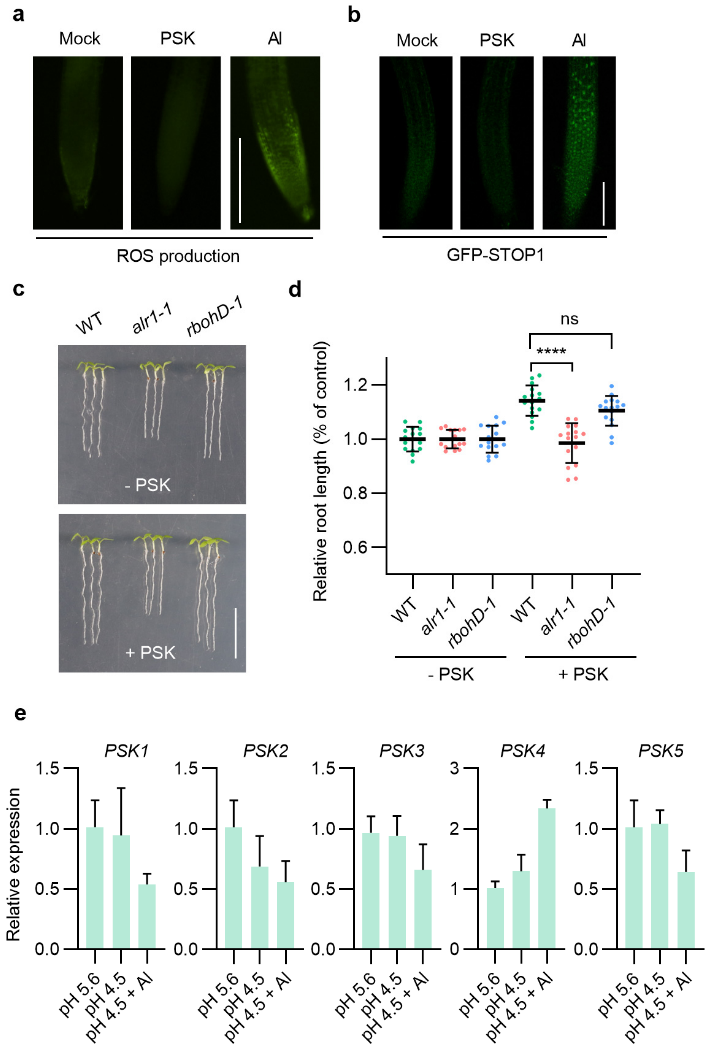

Supplementary information, Fig. S13 Al signaling is separate from PSK signaling. a ROS visual signals in roots under control, PSK (100 nM) and Al (15 μM) treatments for 10 min. **b** GFP-STOP1 fluorescence signals in roots. Bars = 100 μm. **c** Root growth of indicated genotypes under control and PSK (0.1 μM) treatment for 7 days (bar = 1 cm). **d** Quantification of relative root growth in (**c**) (n = 15-16). The average length of each genotype was set to 100%, and the relative root length was expressed as percentage (root length with treatment/root length with control×100). **e** Expression analysis of *PSK* genes in roots under indicated treatments for 6 h (n = 3). Data were analyzed by unpaired t test (**d**) (ns indicates non-significance, *****P*<0.0001).
