# Supplementary material for: The LRR receptor-like kinase ALR1 is a plant aluminum ion sensor
Source: Cell Res. 2024 Jan 10;34(4):281–94. doi: 10.1038/s41422-023-00915-y (PMC10978910; doi:10.1038/s41422-023-00915-y)
Supplement: Supplementary file 14 — Table S1 List of ALR1 interactors identified by split-ubiquitin membrane yeast two-hybrid assay [file 41422_2023_915_MOESM14_ESM.pdf]

Supplementary information, Table S1. List of ALR1 interactors identified by split-ubiquitin membrane yeast two-hybrid assay

Gene ID	Protein
AT5G47910	NADPH/respiratory burst oxidase protein D (RbohD)
AT3G05530	RPT5A（components of 26S proteasome）
AT4G01850	S-adenosylmethionine synthetase 2 (SAM-2)
AT3G53260	Phenylalanine ammonia-lyase 2 (PAL2)
AT3G27430	PBB1（20S Proteasome Gene Family）
AT5G28770	bZIP transcription factor family protein -AtbZIP63
AT3G16420	PYK10-binding protein 1 (PBP1)
AT1G20020	Ferredoxin-NADP[+]-oxidoreductase 2 (FNR2)
AT5G22120	Coiled-coil protein mRNA
AT3G54390	Sequence-specific DNA binding transcription factor mRNA
AT4G30240	t-SNARE family protein
AT1G25230	Transmembrane protein mRNA
AT4G00330	CRCK2 calmodulin-binding receptor-like cytoplasmic kinase 2
AT1G03030	P-loop containing nucleoside triphosphate hydrolases superfamily protein
AT1G51440	Alpha/beta-Hydrolases superfamily protein
AT1G05940	Cationic amino acid transporter 9 (CAT9)
AT3G13160	Tetratricopeptide repeat (TPR)-like superfamily protein
AT5G55220	Trigger factor type chaperone family protein
