# Supplementary material for: The LRR receptor-like kinase ALR1 is a plant aluminum ion sensor
Source: Cell Res. 2024 Jan 10;34(4):281–94. doi: 10.1038/s41422-023-00915-y (PMC10978910; doi:10.1038/s41422-023-00915-y)
Supplement: Supplementary file 15 — Table S2 Sequence of primers used in this study [file 41422_2023_915_MOESM15_ESM.pdf]

Supplementary information, Table S2. Sequence of primers used in this study

Primer	5'-3'sequence	Purpose	Vector
LBb1.3	ATTTTGCCGATTTCGGAAC	Genotyping	
PSKR1-LP	CTCGCTTTCTGGTATGACGAG	Genotyping	
PSKR1-RP	TCCGAAACTATACACATCGCC	Genotyping	
BAK1-LP	CATGACATCATCATCATTCGC	Genotyping	
BAK1-RP	ATTTTGCAGTTTTGCCAACAC	Genotyping	
SERK1-LP	ACTGAAGGAAGAGCGAACTCC	Genotyping	
SERK1-RP	TTGGACCAGATAACTCAACGG	Genotyping	
SERK2-LP	AGTGAAGAGCGAGAAGGAACC	Genotyping	
SERK2-RP	AAGGCTTAGGCTTTTGTTTGG	Genotyping	
PSKR2-LP	GAGAACTTGTTGGAGCTCACG	Genotyping	
PSKR2-RP	TTTTGGGATGTGAGCGTTTAG	Genotyping	
rbohD-1-LP	GCATGTCCACTCTTTTTAGCG	Genotyping	
rbohD-1-RP	CGAGTCGTCCCTGATGTCTAG	Genotyping	
STOP1-LP	CCACCAAACGTGAAGAGAGAG	Genotyping	
STOP1-RP	ATGAAGCACAGGACCATGATC	Genotyping	
dspm11	GGTGCAGCAAAACCCACACTTTTACTTC	Genotyping	
rbohD-2-R	GGATACTGATCATAGGCGTGGCTCCAATGCC GAGACCTAC	Genotyping	
qALMT1-F	TCTCCGTAGGGGCTACACTT	RT-PCR	
qALMT1-R	ACTGTTGCACCCGACAATCT	RT-PCR	
qMATE-F	GTAGCTGGCCAGGCAATACTAGC	RT-PCR	
qMATE-R	GCCACAAACGGAAGTCCTATGC	RT-PCR	
qSTOP1-F	TTCGATTTGCCTAAGCCGGT	RT-PCR	
qSTOP1-R	TCTCATACGAACCGGGAGGA	RT-PCR	
qRbohD-F	TCCACGCACTCAAAGGTCTC	RT-PCR	
qRbohD-R	CGTTGGAATCAGCGGAGAGT	RT-PCR	
qPSKR1-F	CATGAGAGTCGAGCAAGCGA	RT-PCR	
qPSKR1-R	TTGGCCTCTGTTTCGGGTTT	RT-PCR	
qPSK1-F	AGTAGCTGCTCACACCGATT	RT-PCR	
qPSK1-R	AGTAGCTGCTCACACCGATT	RT-PCR	
qPSK2-F	CAAACGTCTCCGCTTTGCTC	RT-PCR	
qPSK2-R	CAAACGTCTCCGCTTTGCTC	RT-PCR	
qPSK3-F	CTTGTGCCTGGCAGTTCTCT	RT-PCR	
qPSK3-R	ACTGAGTCCTCTTCCACTGA	RT-PCR	
qPSK4-F	CTACGCTAACCTACGCAGCA	RT-PCR	
qPSK4-R	CTCCAATTCCGTTGCAGCTTT	RT-PCR	
qPSK5-F	TCTGCTCCACGCTAACACAC	RT-PCR	
qPSK5-R	TCTTCTTCTCCAACACCTTCACA	RT-PCR	
UBQ10-F	GAAACCACCACGAAGAC	RT-PCR	
UBQ10-R	ATCACCCTTGAAGTGGA	RT-PCR	

pPSKR1-SacI-F	GCGAGCTCACCATTGTGACTATCTTTCTTCTG	Cloning	pCAMBIA1301
pPSKR1-KpnI-R	GCGGTACCGCAGTTCAAGAACAGAGGAAG	Cloning	pCAMBIA1301
PSKR1-BamHI-F	GCGGATCCATGCGTGTTCATCGTTTTTGT	Cloning	pCAMBIA1301
PSKR1CD-BamHI-F	GCGGATCCATGCGTGCTCGTAGACGGTCAGG AG	Cloning	pCAMBIA1301
PSKR1-SalI-R	GCGTCGACGACATCATCAAGCCAAGAGACT	Cloning	pCAMBIA1301
pRbohD-SacI-F	GCGAGCTCTATAAGCAAAGCCTTTTGTCGAA A	Cloning	pCAMBIA1301
pRbohD-KpnI-R	GCGGTACCCGAATTCGAGAAACCAAAAAGAT C	Cloning	pCAMBIA1301
cLUC-RbohD-KpnI-F	GATCTCGTACGCGTCCCGGGGCGGTACC ATGAAAATGAGACGAGGCAATTCAAG	Cloning	pCAMBIA1300-35S-cLUC
cLUC-RbohD-SalI-F	GATACGAACGAAAGCTCTGCAGGTCGAC CTAGAAGTTCTCTTTGTGGAAGTC	Cloning	pCAMBIA1300-35S-cLUC
cLUC-RbohA-KpnI-F	GATCTCGTACGCGTCCCGGGGCGGTACC ATGATGAATCGAAGTGAAATGC	Cloning	pCAMBIA1300-35S-cLUC
cLUC-RbohA-SalI-F	GATACGAACGAAAGCTCTGCAGGTCGAC TTAGAAATTCTCTTTATGGAAG	Cloning	pCAMBIA1300-35S-cLUC
cLUC-RbohE-KpnI-F	GATCTCGTACGCGTCCCGGGGCGGTACC ATGAAGTTATCGCCTCTGAGTTTC	Cloning	pCAMBIA1300-35S-cLUC
cLUC-RbohE-SalI-F	GATACGAACGAAAGCTCTGCAGGTCGAC TTAGAAATGCTCCTTATGGAACT	Cloning	pCAMBIA1300-35S-cLUC
nLUC-PSKR1-KpnI-F	GAGAACACGGGGGACGAGCTCGGTACC ATG CGTGTTCATCGTTTTTGTGTG	Cloning	pCAMBIA1300-35S-nLUC
nLUC-PSKR1-SalI-R	GACGCGTACGAGATCTGGTCGACGACATCAT CAAGCCAAGA	Cloning	pCAMBIA1300-35S-nLUC
TF-PSKR1CD-KpnI-F	GAAGGTAGGCATATGGAGCTCGGTACC CGTGCTCGTAGACGGTCAGGAGAAG	Cloning	pCold-TF
TF-PSKR1CD-SalI-R	TACCTATCTAGACTGCAGGTCGAC CTAGACATCATCAAGCCAAGAGACTAACTG	Cloning	pCold-TF
TF-BAK1CD-KpnI-F	GGTAGGCATATGGAGCTCGGTACC ATGACTCCCCTTCCTGCATCTCCAC	Cloning	pCold-TF
TF-BAK1CD-SalI-R	TACCTATCTAGACTGCAGGTCGAC TTATCTTGGACCCGAGGGGTATTCGT	Cloning	pCold-TF
TF-PSKR1ED-KpnI-F	GAAGGTAGGCATATGGAGCTCGGTACC ATGCAGACCACCTCCAGGTGCCA	Cloning	pCold-TF
TF-PSKR1ED-SalI-R	TACCTATCTAGACTGCAGGTCGAC TCAAATGTCACCTCCTCTGCTTCTTCTT	Cloning	pCold-TF
TF-RAE1-KpnI-F	GAAGGTAGGCATATGGAGCTCGGTACC ATGAAGAAGGTTAAACAGATTCGTGTC	Cloning	pCold-TF
TF-RAE1-SalI-R	TACCTATCTAGACTGCAGGTCGAC TTAAGGCGCCATCTCTTCCAGCTGTTGC	Cloning	pCold-TF
TF-STOP1-KpnI-F	TACCTATCTAGACTGCAGGTCGAC ATGGAAACTGAAGACGATTTGTGCAAC	Cloning	pCold-TF
TF-STOP1-SalI-R	TACCTATCTAGACTGCAGGTCGAC TTAGAGACTAGTATCTGAAACAGACTCAC	Cloning	pCold-TF

TF-RbohDN-KpnI-F	GGTAGGCATATGGAGCTCGGTACC ATGAAAATGAGACGAGGCAATTCAAG	Cloning	pCold-TF
TF-RbohDN-SalI-R	TACCTATCTAGACTGCAGGTCGAC AGTCTCTGCCAATTGTCAAGTATGAAA	Cloning	pCold-TF
Flag-RbohD-KpnI-F	AAAGTCGAGGGGGGGCCCGGTACC ATGAAAATGAGACGAGGCAATTC	Cloning	pCAMBIA1300-35S-Flag
Flag-RbohD-SalI-R	TACTCGAACCTGCAGGTCGAC CTAGAAGTTCTCTTTGTGGAAGT	Cloning	pCAMBIA1300-35S-Flag
Flag-RAE1-KpnI-F	AAAGTCGAGGGGGGGCCCGGTACC ATGAAGAAGGTTAAACAGATT	Cloning	pCAMBIA1300-35S-Flag
Flag-RAE1-SalI-R	TACTCGAACCTGCAGGTCGAC TTAAGGCGCCATCTCTTCCAG	Cloning	pCAMBIA1300-35S-Flag
pUC35S-Flag-RAE1-F	TCAGCAGTCGAAGAGC ATGAAGAAGGTTAAACAGATTCGTGT	Cloning	pUC35S-Flag/N
pUC35S-Flag-RAE1-R	TTAGCGTGTGAAGAGC AGGCGCCATCTCTTCCAGCT	Cloning	pUC35S-Flag/N
pUC35S-nYFP-PSKR1-F	TCAGCAGTCGAAGAGC ATGCGTGTTCATCGTTTTTGT	Cloning	pUC35S-nYFP
pUC35S-nYFP-PSKR1-R	TTAGCGTGTGAAGAGC GACATCATCAAGCCAAGAGAC	Cloning	pUC35S-nYFP
pUC35S-nYFP-PSKR1CD-F	TCAGCAGTCGAAGAGC ATGCGTGCTCGTAGACGGTCAGGA	Cloning	pUC35S-nYFP
pUC35S-nYFP-PSKR1CD-R	TTAGCGTGTGAAGAGC GACATCATCAAGCCAAGAGAC	Cloning	pUC35S-nYFP
pUC35S-cYFP-RbohD-F	TCAGCAGTCGAAGAGC ATGAAAATGAGACGAGGCAAT	Cloning	pUC35S-cYFP
pUC35S-cYFP-RbohD-R	TTAGCGTGTGAAGAGC GAAGTTCTCTTTGTGGAAGTC	Cloning	pUC35S-cYFP
pUC35S-cYFP-BAK1-F	TCAGCAGTCGAAGAGC ATGGAACGAAGATTAATGATCCC	Cloning	pUC35S-cYFP
pUC35S-cYFP-BAK1-R	TTAGCGTGTGAAGAGC TCTTGGACCCGAGGGGTATTCGT	Cloning	pUC35S-cYFP
pUC35S-cYFP-BAK1ED-F	TCAGCAGTCGAAGAGC ATGGAACGAAGATTAATGATCCC	Cloning	pUC35S-cYFP
pUC35S-cYFP-BAK1ED-R	TTAGCGTGTGAAGAGC CAACTTGGTGTTGGCAAAACTGA	Cloning	pUC35S-cYFP
HBT-35S-GFP-STOP1-BamHI-F	CCTCTCCCCTTGCTCCGTGGATCC ATGGAAACTGAAGACGATTTGTGCAAC	Cloning	HBT-35S-GFP
HBT-35S-GFP-STOP1-StuI-R	TTCTTCTCCCTTGCTCATAGGCCT GAGACTAGTATCTGAAACAGACTCAC	Cloning	HBT-35S-GFP
pPR3-RbohD-Sfi-F	ATGGCCATTACGGCCCGGATGAAAATGAGAC GAGGCAATTCA	Cloning	pPR3-N
pPR3-RbohD-Sfi-R	ATGGCCGAGGCGGCCCTAGAAGTTCTCTTTG TGGAAGTCAAA	Cloning	pPR3-N
pPR3-STOP1-Sfi-F	ATGGCCATTACGGCCCGG ATGGAAACTGAAGACGATTTGTGCAAC	Cloning	pPR3-N

pPR3-STOP1-Sfi-R	ATGGCCGAGGCGGCC TTAGAGACTAGTATCTGAAACAGACTCAC	Cloning	pPR3-N
STE-PSKR1-Sfi-F	ATGGCCATTACGGCCCGG ATGCGTGTTCATCGTTTTTGT	Cloning	pBT3-STE
STE-PSKR1-Sfi-R	ATGGCCGAGGCGGCC GACATCATCAAGCCAAGAGACT	Cloning	pBT3-STE
RbohD-S39D-F	TTTGACGGTCCGCTTGGCCGGCCTAAAC	Cloning/Mutagenesis	pCAMBIA1301
RbohD-S39D-R	GGCACCACGGTCGCTAGCGATGCTCTCC	Cloning/Mutagenesis	pCAMBIA1301
RbohD-S39A-F	TTTGCCGGTCCGCTTGGCCGGCCTAAAC	Cloning/Mutagenesis	pCold-TF
RbohD-S39A-R	GGCACCACGGTCGCTAGCGATGCTCTCC	Cloning/Mutagenesis	pCold-TF
RbohD-S152A-F	TTCTCTAGACGTCCCGCCCCGGCCGTG	Cloning/Mutagenesis	pCold-TF
RbohD-S152A-R	CACGCGGCGGAGCTCGCGGGAGGCGTT	Cloning/Mutagenesis	pCold-TF
PSKR1-C742A-F	GGCGGTGGCGGGTTTGGTATGGTTTACAA	Cloning/Mutagenesis	pCold-TF
PSKR1-C742A-R	AATGATGTTAGCTTGATCAAAACTATT	Cloning/Mutagenesis	pCold-TF
PSKR1-C768A-F	GATGGCGGTCAAATCGAAAGAGAAT	Cloning/Mutagenesis	pCold-TF
PSKR1-C768A-R	ACCGGATAACTTCTTGATCGCAACTTT	Cloning/Mutagenesis	pCold-TF
PSKR1-C852A-F	GGGGGTGATCCTCATATCTTACACCG	Cloning/Mutagenesis	pCold-TF
PSKR1-C852A-R	TTCATGCAAGTAAAGTAACCCTTTTGC	Cloning/Mutagenesis	pCold-TF
PSKR1-K762R-F	ATCAGGAAGTTATCCGGTGATTGC	Cloning/Mutagenesis	pCold-TF/pCAMBIA1301
PSKR1-K762R-R	CGCAACTTTCTTACCGTCTGGTAACG	Cloning/Mutagenesis	pCold-TF/pCAMBIA1301
PSKR1-C939/944A-F	GGGTAAACCGAAAGGGGGTAGGGATCT	Cloning/Mutagenesis	pCold-TF/pCAMBIA1301
PSKR1-C939/944A-R	ATATCCACCGGTCTTTTATCGGTTAAA	Cloning/Mutagenesis	pCold-TF/pCAMBIA1301
PSKR1-C985/987A-F	GCTGGTTTAGGTTTAAGCGAAAACCCG	Cloning/Mutagenesis	pCold-TF/pCAMBIA1301
PSKR1-C985/987A-R	AATCTCGAGAACCCGAAACATCTCTT	Cloning/Mutagenesis	pCold-TF/pCAMBIA1301
PSKR1-R300A-F	GGCGAACAATTCTTTATCGGGT	Cloning/Mutagenesis	pCAMBIA1301
PSKR1-R300A-R	CAAGTTAAGCAGATTCAAACTCGGT	Cloning/Mutagenesis	pCAMBIA1301
PSKR1-F506A-F	CGCTATGAAAAGAAACGAGAGC	Cloning/Mutagenesis	pCAMBIA1301
PSKR1-F506A-R	GCTCTCGTTTCTTTTCATAAAG	Cloning/Mutagenesis	pCAMBIA1301
STOP1-C27A-F	CTCTGGGAATTCGACATTCGC	Cloning/Mutagenesis	HBT-35S-GFP
STOP1-C27A-R	TCTGATGAACCCGGCTCTCG	Cloning/Mutagenesis	HBT-35S-GFP
STOP1-C185A-F	CTCTGGTGCTCGAGAGTTCGATT	Cloning/Mutagenesis	HBT-35S-GFP
STOP1-C185A-R	TTGTTATTGTTCTGACTCTGTGAAGCA	Cloning/Mutagenesis	HBT-35S-GFP
STOP1-C335A-F	TTCCAGCCGGTGCCATACC	Cloning/Mutagenesis	HBT-35S-GFP
STOP1-C335A-R	GTGAAGCTTTTATCGCAGTGGGT	Cloning/Mutagenesis	HBT-35S-GFP
RAE1-C160A-F	GCCAAGATGCTGACGGACATGG	Cloning/Mutagenesis	pUC35S-Flag/N
RAE1-C160A-R	TCTGCCCAGCTTCAGCCTCTCC	Cloning/Mutagenesis	pUC35S-Flag/N
RAE1-C218A-F	GCTTTACATGACATTCTGAAACTTCAACAC	Cloning/Mutagenesis	pUC35S-Flag/N
RAE1-C218A-R	CTTTCCTGTGATCGGCAAGTAGGAG	Cloning/Mutagenesis	pUC35S-Flag/N
RAE1-C262A-F	GCCCAGAATTTAACTCATAGAGGTTTAACC	Cloning/Mutagenesis	pUC35S-Flag/N
RAE1-C262A-R	GCTGGATGCATCAAGCTTCTTC	Cloning/Mutagenesis	pUC35S-Flag/N
RAE1-C288A-F	GCTTCTTCTGTGATATCATTGGATTTTG	Cloning/Mutagenesis	pUC35S-Flag/N
RAE1-C288A-R	GTGTGATAGATCAAGTCGCTGAAG	Cloning/Mutagenesis	pUC35S-Flag/N
RAE1-C339A-F	GCCGTGAGCGTAACTGATGAAGGTC	Cloning/Mutagenesis	pUC35S-Flag/N
RAE1-C339A-R	TTTGCTTAGGCTAACCTCTTTCAGGG	Cloning/Mutagenesis	pUC35S-Flag/N

RAE1-C364A-F	GCTTGCCGGAAACTAAGTAGAGTTTC	Cloning/Mutagenesis	pUC35S-Flag/N
RAE1-C364A-R	TGTGATGTCAAGTTTTCTGAGGTCT	Cloning/Mutagenesis	pUC35S-Flag/N
RAE1-C365A-F	GCCCGGAAACTAAGTAGAGTTTCAATC	Cloning/Mutagenesis	pUC35S-Flag/N
RAE1-C365A-R	ACATGTGATGTCAAGTTTTCTGAGG	Cloning/Mutagenesis	pUC35S-Flag/N
RAE1-C391A-F	GCTTCTCTTGTTTCCAGAGAAGCC	Cloning/Mutagenesis	pUC35S-Flag/N
RAE1-C391A-R	AGACTCCATCTTCAAAGAGACTAGTAA	Cloning/Mutagenesis	pUC35S-Flag/N
RAE1-C507A-F	GCCTCGTTGTTACAAACATTCGAGAG	Cloning/Mutagenesis	pUC35S-Flag/N
RAE1-C507A-R	TTTGGACAATGAAACCAGGGACTTG	Cloning/Mutagenesis	pUC35S-Flag/N
